# *Tripterygium wilfordii Hook.f* induced kidney injury through mediating inflammation via PI3K-Akt/HIF-1/TNF signaling pathway: A study of network toxicology and molecular docking

**DOI:** 10.1097/MD.0000000000036968

**Published:** 2024-02-09

**Authors:** Shuo Yang, Mengmeng Wang, Zhongming Li, Xiangjia Luan, Yanan Yu, Junjie Jiang, Yuanyuan Li, Yanming Xie, Lianxin Wang

**Affiliations:** a Institute of Basic Research in Clinical Medicine, China Academy of Chinese Medical Sciences, Beijing, China; b School of Artificial Intelligence, Beijing University of Posts and Telecommunications (BUPT), Beijing, China; c Guang’anmen Hospital, China Academy of Chinese Medical Sciences, Beijing, China.

**Keywords:** drug induced kidney injury, inflammation, molecular docking, network toxicology, PI3K-Akt signaling pathway, *Tripterygium wilfordii Hook.f* (TwHF), Triptonide

## Abstract

We intend to explore potential mechanisms of *Tripterygium wilfordii Hook.f* (TwHF) induced kidney injury (KI) using the methods of network toxicology and molecular docking. We determined TwHF potential compounds with its targets and KI targets, obtained the TwHF induced KI targets after intersecting targets of TwHF and KI. Then we conducted protein-protein interaction (PPI) network, gene expression analysis, gene ontology (GO) function and Kyoto encyclopedia of genes and genomes (KEGG) pathway enrichment analysis to explore the mechanism of TwHF-induced KI. Finally we conducted molecular docking to verify the core toxic compounds and the targets. We obtained 12 TwHF toxic compounds and 62 TwHF-induced KI targets. PPI network, gene expression analysis and GO function enrichment analysis unveiled the key biological process and suggested the mechanism of TwHF-induced KI might be associated with inflammation, immune response, hypoxia as well as oxidative stress. KEGG pathway enrichment analysis indicated PI3K-Akt signaling pathway, HIF-1 signaling pathway and TNF signaling pathway were key signaling pathways of TwHF induced KI. Molecular docking showed that the binding energy of core targets and toxic compounds was all less than −6.5 kcal/mol that verified the screening ability of network pharmacology and provided evidence for modifying TwHF toxic compounds structure. Through the study, we unveiled the mechanism of TwHF induce KI that TwHF might activate PI3K-Akt signaling pathway as well as TNF signaling pathway to progress renal inflammation, mediate hypoxia via HIF-1 signaling pathway to accelerate inflammatory processes, and also provided a theoretical basis for modifying TwHF toxic compounds structure as well as supported the follow-up research.

## 1. Introduction

*Tripterygium wilfordii Hook.f* (TwHF), known as Lei Gong Teng in Chinese, is a traditional Chinese herbal medicine and first recorded in the traditional Chinese medicine (TCM) classics - *shennong bencao jing*. TwHF belongs to the *Tripterygium wilfordii* family, main components include sesquiterpenes, diterpenes, triterpenes and alkaloids^[1,2]^ and main medicinal part is root. TwHF has rich human-used experience in clinic practice, and is mostly used to treat Immune system diseases such as rheumatoid arthritis,^[3,4]^ kidney disease,^[5,6]^ systemic lupus erythematosus^[7,8]^ and so on. Owing to its anti-inflammatory and immune-regulated effects, TwHF was regarded as Chinese medical hormone.^[5,8]^ In addition, TwHF also has broad-spectrum anticancer activities which are effective for various cancers.^[9]^ With the development of technology, TwHF preparations have been developed and used widely for different diseases.^[10,11]^ While the therapeutic effects of these preparations are significant, the toxic doses of TwHF are close to the therapeutic doses which led to a focus on its safety.^[8,12]^ Apart from common adverse reactions such as intestinal toxicity, reproductive toxicity, hepatotoxicity and cutaneous toxicity, TwHF also can contribute to toxicity and injury of kidney.^[13,14]^ Studies showed that high concentration of TwHF extracts (1.5 mg/mL) had potential inhibition of ConA-stimulated lymphocyte proliferation for African green monkey kidney COS-7 cells,^[15]^ and Tripterygium glycosides from TwHF would result in Segment-specific proximal tubule injury to further kidney injury.^[16]^

Drug induced kidney injury (DIKI), is defined as a new onset of kidney injury or the worsening of an existing kidney injury due to drug administration.^[17]^ DIKI encompasses acute kidney injury (AKI), glomerular disorders, tubular dysfunction, and nephrolithiasis. Statistics indicate that DIKI is a major contributing factor in approximately 60% of AKI cases in hospitalized patients.^[18]^ DIKI can be driven by renal tubular cytotoxicity, altered glomerular hemodynamics, inflammation and so on.^[19]^ It has been validated that TwHF could result in KI, while it is unclear which specific compounds within TwHF can easily cause KI and what toxicology mechanism of TwHF induced KI. These need to be solved. Meanwhile, the toxin reduction and efficiency enhancement are goal of TwHF preparations, so the research on TwHF potential toxicity is essential too.

Network toxicology, derived and developed from network pharmacology, is an important method for medication toxicity studies.^[20]^ Through analyzing the network connection between organism and toxic compounds that contribute to abnormal biological functions, we can explain the toxicological mechanisms of toxic compounds.^[21]^ Molecular docking is a theoretical simulation technology which often used to study inter-molecular interactions and predict their binding modes and affinities for further new drug discovery.^[22,23]^

**Figure 1.**
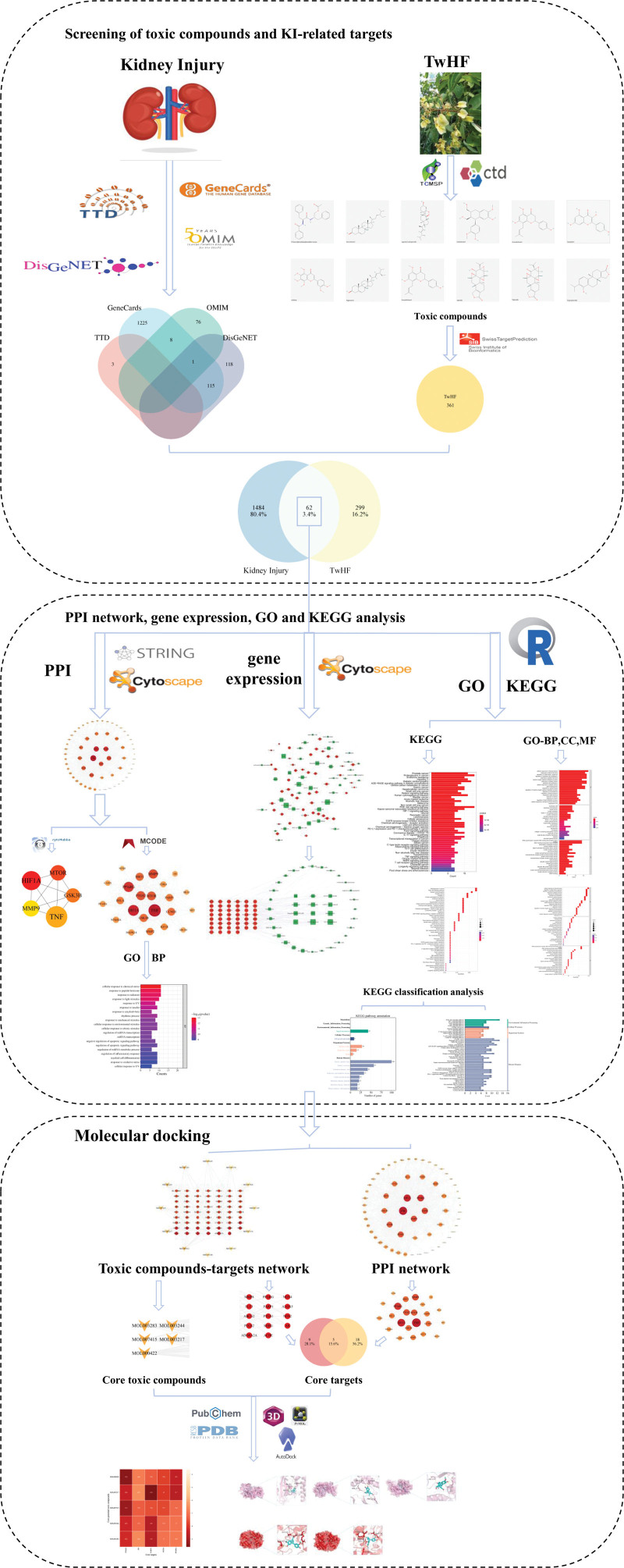
The research diagram.

In the study, we conducted a multi-compounds, multi-targets and multi-approaches study using network toxicology and molecular docking to determine the main toxic compounds of TwHF induced KI and explain the toxicology mechanism of TwHF-induced KI. The research design is as follows in Figure 1.

## 2. Materials and methods

### 2.1. Acquirement of TwHF toxic compounds

We searched Traditional Chinese Medicine Systems Pharmacology (TCMSP) Database^[24]^ (https://old.tcmsp-e.com/tcmsp.php) to obtain all compounds of TwHF, then filtered bioactive compounds with oral bioavailability (OB) over 30% and drug-likeness over 0.18.^[25]^ Next we inputted these bioactive compounds into Comparative Toxicogenomics Database^[26]^ (CTD, https://ctdbase.org/) to select potential toxic compounds within TwHF. Further, we determined toxic compounds pharmacokinetics from Swiss ADME platform^[27]^ (http://www.swissadme.ch/) for toxic mechanism analysis.

### 2.2. Establishment of TwHF induced kidney injury targets

We inputted toxic compounds SMILES number into Swiss Target Prediction online platform^[28]^ (http://www.swisstargetprediction.ch/) to get targets of each toxic compound, then merged them all and removed duplicated to get TwHF toxic compounds targets. Next we searched GeneCards databases^[29]^ (http://www.genecards.org/), OMIM database^[30]^ (http://www.omim.org/), DisGeNET database^[31]^ (https://www.disgenet.org/) and Therapeutic Target Database^[32]^ (TTD, http://db.idrblab.net/ttd/) using keyword of kidney injury to get relevant targets. And after merging all genes and removing duplicated, we obtained the kidney injury relevant targets. Finally we determined the TwHF induced KI targets by taking intersections using Venn diagram.

### 2.3. Constructions of networks and screening of core toxic compounds and targets

We inputted TwHF induced KI targets into STRING database^[33]^ (http://string-db.org) to obtain Protein-protein interaction (PPI) information. After visualizing the PPI network using Cytoscape_3.9.1, we also used plug-ins such as cytoNCA, MCODE and cytoHubba to obtain topological parameters, find potential functional modules and determine hub genes for core targets screening. In addition, with toxic compounds and targets as nodes, their interaction information as edges, we constructed toxic compounds-targets network, then we screened core toxic compounds using Degree value.

### 2.4. Gene expression analysis of targets

We obtained gene expression level of each target from BioGPS database^[34]^ (https://biogps.org). The default datasets was GeneAtlas U133A, gcrma. We selected relevant organ/tissue/cell with expression levels 3 times higher than the median as the specific expression results for the gene. And we constructed and visualized the target-specific expression network through Cytoscape_3.9.1.

### 2.5. GO function and KEGG pathway enrichment analysis of targets

We used clusterProfiler R package to process Gene Ontology (GO) function and Kyoto Encyclopedia of Genes and Genomes (KEGG) pathway enrichment analyses of TwHF induced KI targets. We filtered results by a significant threshold of *P* < .01, then visualized using the ggplot2 R package. Top 20 enrichment results of biological process (BP), molecular function (MF) and cellular component (CC) from GO function were used to analyze. And top 50 enrichment results of KEGG pathway were used to analyze. In addition, we categorized and summarized KEGG pathways results based on the 6 classification from KEGG PATHWAY Database (https://www.genome.jp/kegg/) to further analysis.

### 2.6. Molecular docking of core compounds and targets

We obtained 2D structure of each core toxic compound through PubChem database (https://pubchem.ncbi.nlm.nih.gov/) and then used Chemdraw_20.0 to drew 3D structure, minimized the structural energy and saved as.mol2 format. Meanwhile, we obtained 3D structure of each core target from Uniprot database (http://www.UniProt.org/) and converted to.pdb format, then we used PyMOL_2.3.0 to deal with water and other small molecular ligands for ligand preparation. After above preparations are completed, putting 5 potential toxic compounds as ligand molecules and 5 targets as receptor, we used Autodock Vina_1.1.2 software to perform molecular docking. Finally, we used PyMOL software for analysis and visualization.

## 3. Results

### 3.1. Acquirement of TwHF toxic compounds

We obtained 144 TwHF compounds (Supplementary Table 1, http://links.lww.com/MD/L485), filtered 51 bioactive compounds, and got 12 potential toxic compounds associated with diseases really. The basic information of toxic compounds are in Table 1. In addition, we inputted these toxic compounds into Swiss ADME platform and got their pharmacokinetic information (Table 2). And it is clear that most toxic compounds had high gastrointestinal absorption capacity and all of them had low skin permeability.

**Table 1:** Basic information of 12 toxic compounds.

MOL ID	Molecule name	MW	Hdon	OB (%)	Caco-2	BBB	DL
MOL000358	beta-sitosterol	414.79	1	36.91	1.32	0.99	0.75
MOL000422	kaempferol	286.25	4	41.88	0.26	−0.55	0.24
MOL000449	Stigmasterol	412.77	1	43.83	1.44	1.00	0.76
MOL003187	triptolide	360.44	1	51.29	0.25	−0.19	0.68
MOL003188	Tripchlorolide	396.90	2	78.72	0.16	−0.31	0.72
MOL003196	Tryptophenolide	312.44	1	48.5	1.11	0.69	0.44
MOL003217	Isoxanthohumol	354.43	2	56.81	0.76	−0.01	0.39
MOL003244	Triptonide	358.42	0	68.45	0.15	−0.30	0.68
MOL003283	Isolariciresinol ((2R,3R,4S)-4-(4-hydroxy-3-methoxy-phenyl)-7-methoxy-2,3-dimethylol-tetralin-6-ol)	360.44	4	66.51	−0.20	−1.17	0.39
MOL005828	nobiletin	402.43	0	61.67	1.05	−0.08	0.52
MOL007415	N-benzoylphenylalanylphenylalinol acetate ([(2S)-2-[[(2S)-2-(benzoylamino)-3-phenylpropanoyl]amino]-3-phenylpropyl] acetate)	444.57	2	58.02	0.32	−0.26	0.52
MOL011169	Peroxyergosterol	428.72	1	44.39	0.86	0.43	0.82

BBB = blood-brain barrier, DL = drug-likeness, MW = molecular weight, OB = oral relative bioavailability.

**Table 2:** Pharmacokinetics of 12 toxic compounds.

Mol ID	GI absorption	BBB permeant	CYP1A2 inhibitor	CYP2C19 inhibitor	CYP2C9 inhibitor	CYP2D6 inhibitor	CYP3A4 inhibitor	Log K_p_ (skin permeation)
MOL000358	Low	No	No	No	No	No	No	−2.2cm/s
MOL000422	High	No	Yes	No	No	Yes	Yes	−6.7cm/s
MOL000449	Low	No	No	No	Yes	No	No	−2.74cm/s
MOL003187	High	No	No	No	No	No	No	−8.34cm/s
MOL003188	High	No	No	No	No	No	No	−8.37cm/s
MOL003196	High	Yes	No	Yes	Yes	No	Yes	−5.33cm/s
MOL003217	High	Yes	No	Yes	Yes	No	Yes	−5.56cm/s
MOL003244	High	No	No	No	No	No	No	−8.02cm/s
MOL003283	High	No	No	No	No	Yes	No	−7.04cm/s
MOL005828	High	No	No	No	Yes	No	Yes	−6.62cm/s
MOL007415	High	No	No	Yes	Yes	Yes	Yes	−5.85cm/s
MOL011169	High	No	No	No	No	No	No	−4.15cm/s

GI = gastrointestinal.

### 3.2. Establishment of TwHF induced kidney injury targets

**Figure 2.**
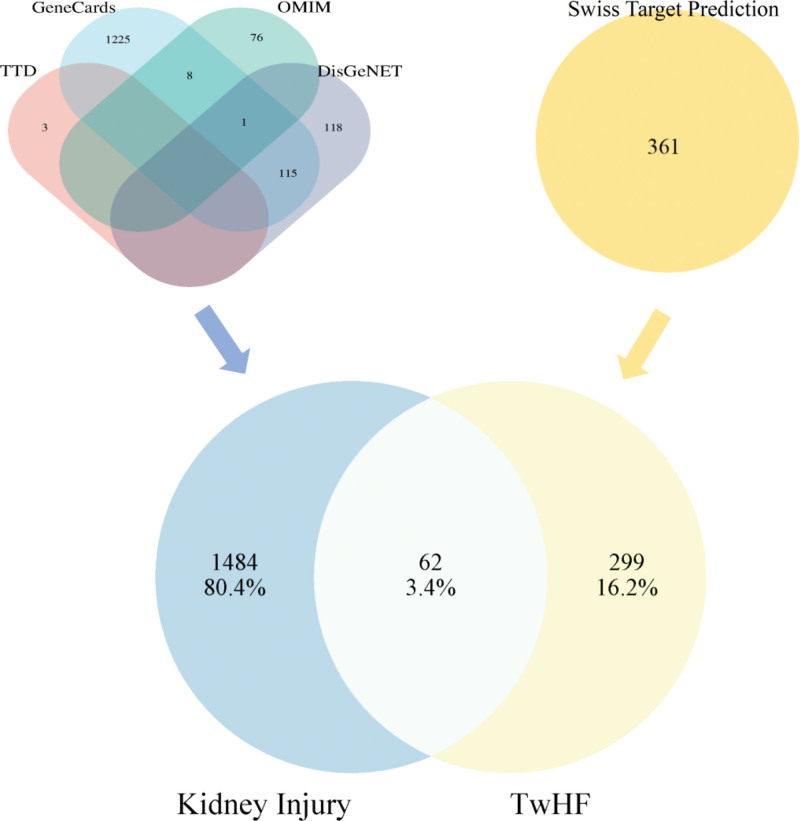
Venn diagram of TwHF induced kidney injury targets. TwHF = Tripterygium wilfordii Hook.f.

We obtained 361 targets of TwHF potential toxic compounds targets (Supplementary Table 2, http://links.lww.com/MD/L486), and 1546 targets relevant to kidney injury from GeneCards (1349, over median), OMIM (85), DisGeNET (234) and TTD (3) databases (Supplementary Table 3, http://links.lww.com/MD/L487). After intersecting, we obtained 62 TwHF induced kidney injury targets (Fig. 2, Supplementary Table 4, http://links.lww.com/MD/L488).

### 3.3. Constructions of networks and screening of core compounds and targets

**Figure 3.**
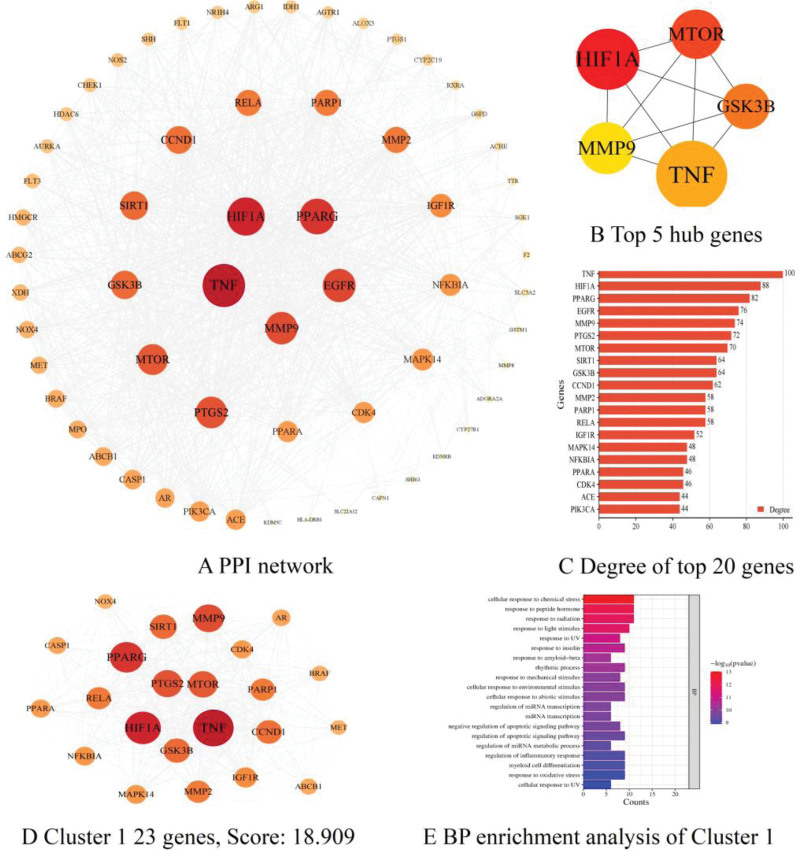
PPI network. PPI = protein-protein interaction.

The PPI network included 61 nodes and 1088 edges (Fig. 3A) with Degree value of 30. Through cytoHubba plug-in, we obtained the top 5 hub genes and they were Hypoxia inducible factor-1 (HIF1A), MTOR, GSK3B, TNF and MMP9 (Fig. 3B). We also ranked the top 20 genes in terms of Degree value, and found that TNF was the highest, HIF1A followed behind, while MTOR was seventh, GSK3B was ninth, and MMP9 was fifth (Fig. 3C). In addition, we used MCODE plug-in to find densely connected regions, and we obtained 2 sub-networks of which cluster 1 scored 18.909 including 23 nodes and 416 edges (Fig. 3D) as well as cluster 2 scored 4 including 5 nodes and 16 edges. Then we further analyzed the biological process from GO function of cluster 1 which revealed that cellular response to chemical stress, response to peptide hormone, negative regulation of apoptotic signaling pathway, regulation of inflammatory response and response to oxidative stress were significantly enriched (Fig. 3E).

**Figure 4.**
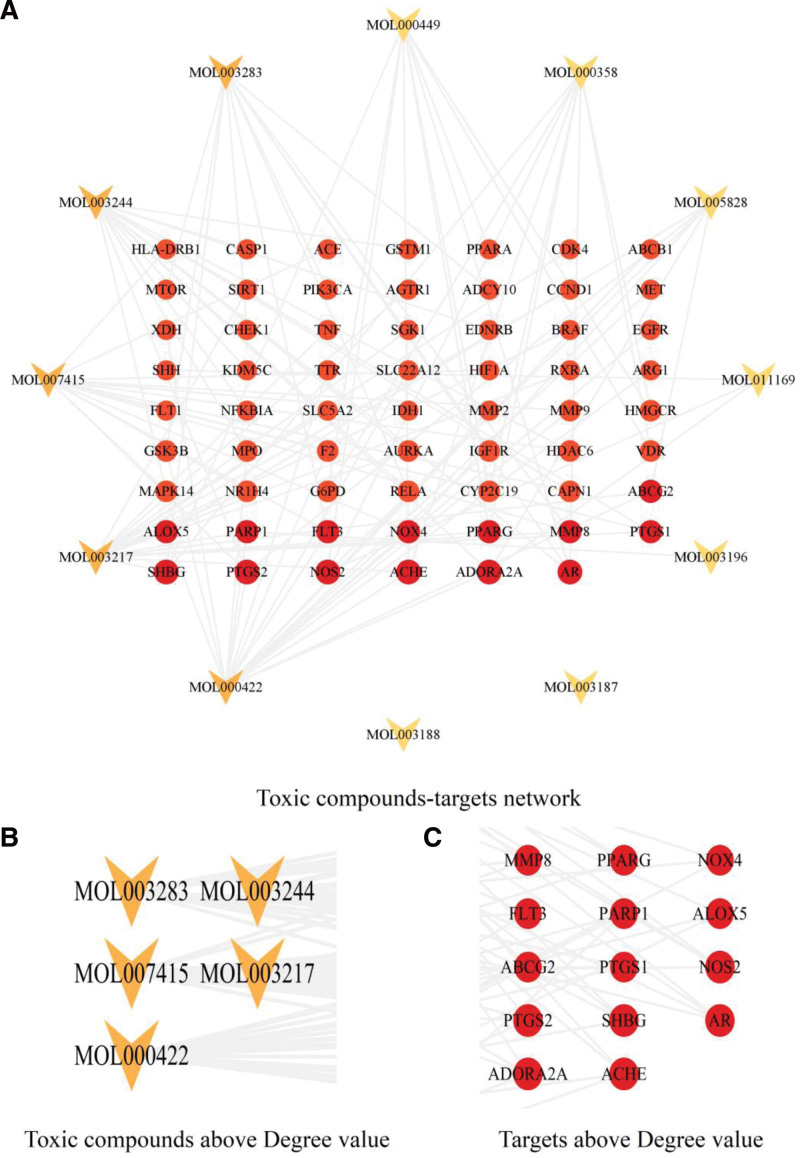
Toxic compounds-targets network. In the network, orange represents Mol ID number of potential toxic compounds, red represents targets.

**Figure 5.**
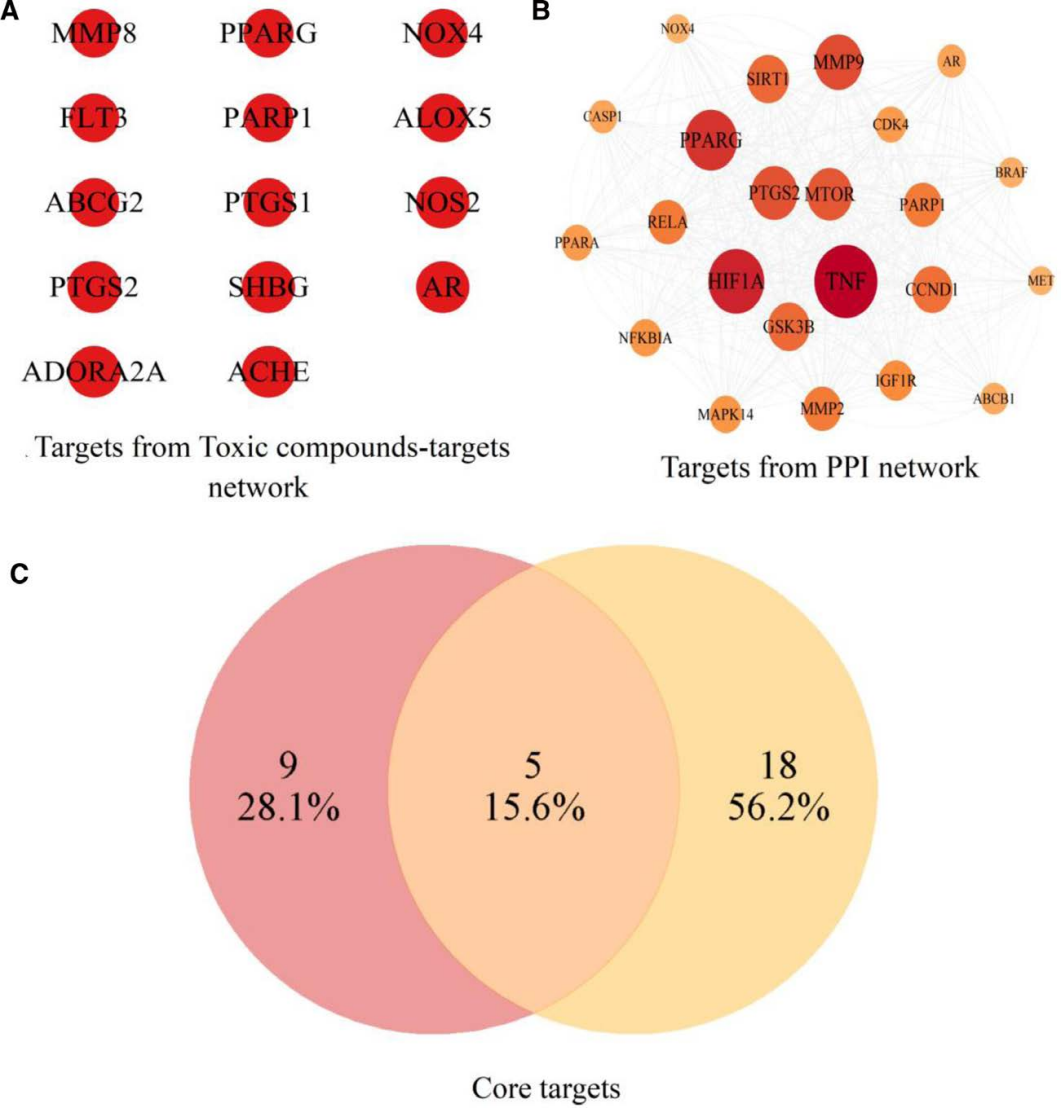
Venn diagram of core targets.

With potential toxic compounds and targets as nodes, their interaction information as edges, we constructed and visualized toxic compounds-targets network (Fig. 4A). Through cytoNCA plug-in, we calculated the Degree value of the network. There were 5 toxic compounds over 5 times of Degree median (Fig. 4B) and 14 targets over Degree median (Fig. 4C). So we selected these 5 toxic compounds as core compounds and they were MOL000422 (kaempferol), MOL003217 (Isoxanthohumol), MOL007415 (N-benzoylphenylalanylphenylalinol acetate), MOL003244 (Triptonide) and MOL003283 (isolariciresinol). To further screening the core targets, we intersected the 14 targets from toxic compounds-targets network with the 23 targets from cluster 1 belonging to PPI network, and obtained the core targets including Prostaglandin endoperoxide synthase 2 (PTGS2), AR, poly (ADP-Ribose) Polymerase 1 (PARP1), NOX4 and PPARG (Fig. 5).

### 3.4. Gene expression analysis of targets

**Figure 6.**
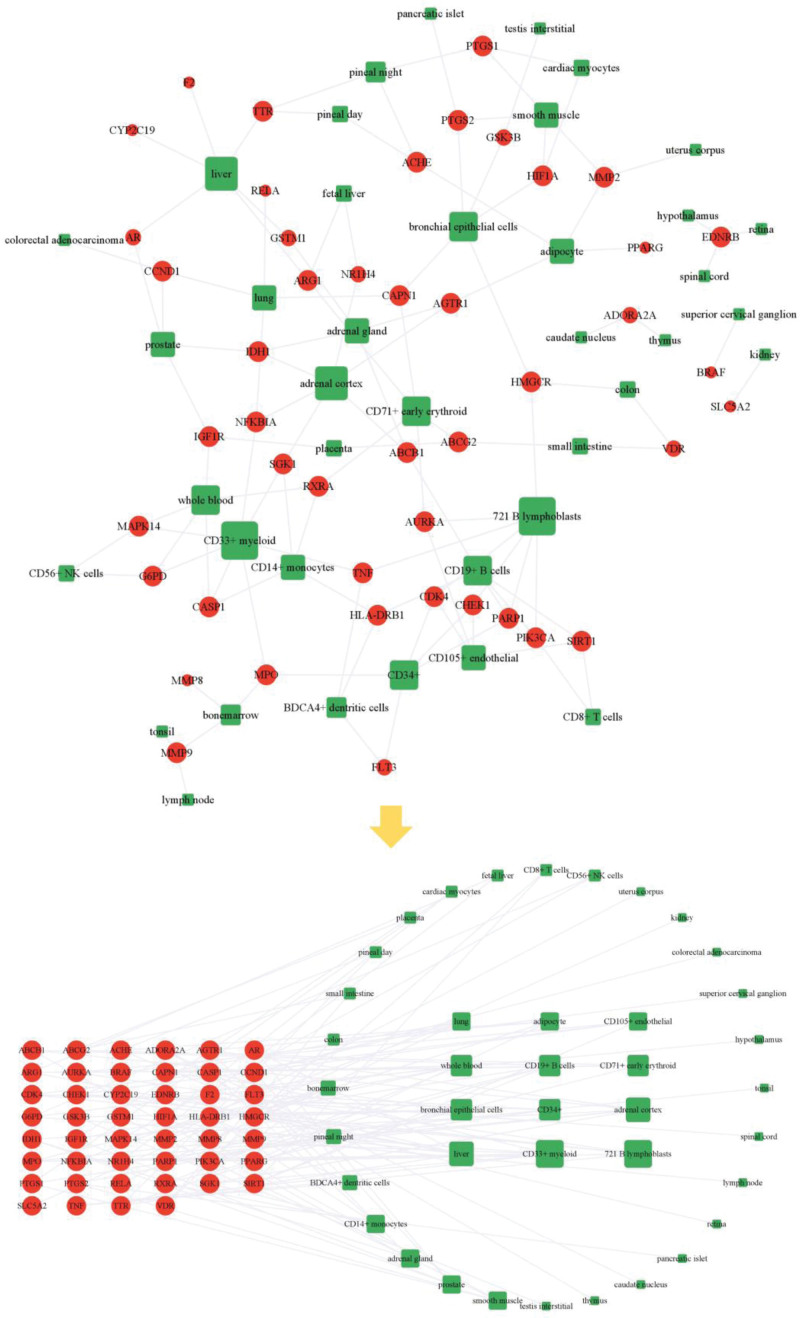
Gene specific expression network. In the network, green represents organ/tissue/cell, red represents targets.

We inputted 62 targets into BioGPS database and obtained the specific expression result of each target (Supplementary Table 5, http://links.lww.com/MD/L489). Then we constructed and visualized the gene specific expression network (Fig. 6). The network showed that immune cells such as 721 B lymphoblasts, CD33 + myeloid, CD34+, CD71 + early erythroid, CD19 + B cells, adipocytes and CD105 + endothelial were higher expressed. In addition, adrenal cortex, liver and lung were higher expressed too.

### 3.5. GO function and KEGG pathway enrichment analysis of targets

**Figure 7.**
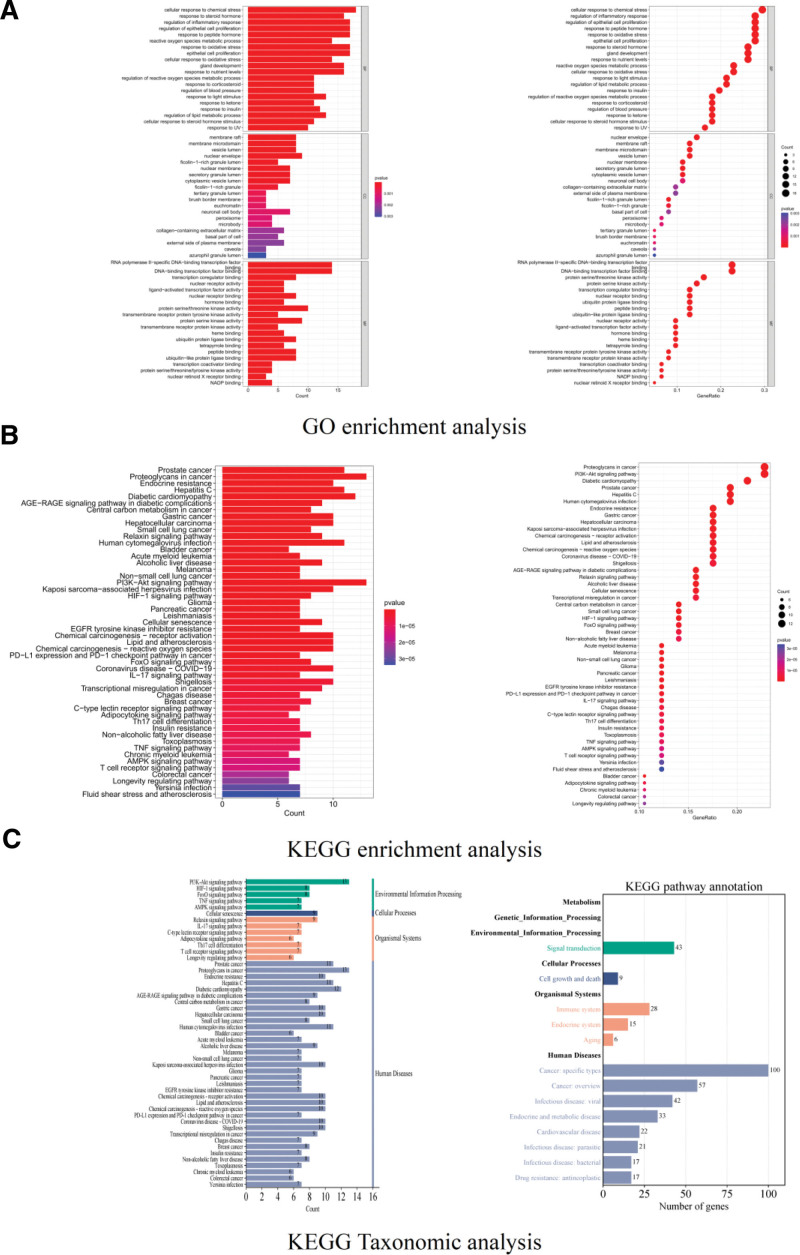
GO function and KEGG pathway enrichment results. GO = gene ontology, KEGG = Kyoto encyclopedia of genes and genomes.

GO function enrichment analysis showed BP, MF and CC results (Fig. 7A) which indicated that BP such as cellular response to chemical stress, regulation of inflammatory, regulation of epithelial cell proliferation, response to peptide hormone and response to oxidative stress were enriched significantly, MF such as RNA polymerase II-specific DNA-binding transcription factor binding, DNA-bindling transcription factor binding, protein serine/threonine kinase activity, protein seine kinase activity and transcription coregulator binding were enriched significantly and CC such as nuclear envelope, membrane raft, membrane microdomain, vesicle lumen, and neuronal membrane were enriched significantly. KEGG pathway enrichment analysis (Fig. 7B) showed that Proteoglycans in cancer, PI3K-Akt signaling pathway, Diabetic cardiomyopathy, Prostate cancer and Hepatitis C were highly enriched. KEGG taxonomic analysis revealed the enrichment pathways and targets of the 6 classifications from KEGG PATHWAY Database. We found that in the section of Environmental Information Processing (Fig. 7C), PI3K-Akt signaling pathway, HIF-1 signaling pathway, FoxO signaling pathway, TNF signaling pathway and AMPK signaling pathway were enriched, and the enriched targets were 43, all belonging to Signal transduction.

### 3.6. Molecular docking of core compounds and targets

**Figure 8.**
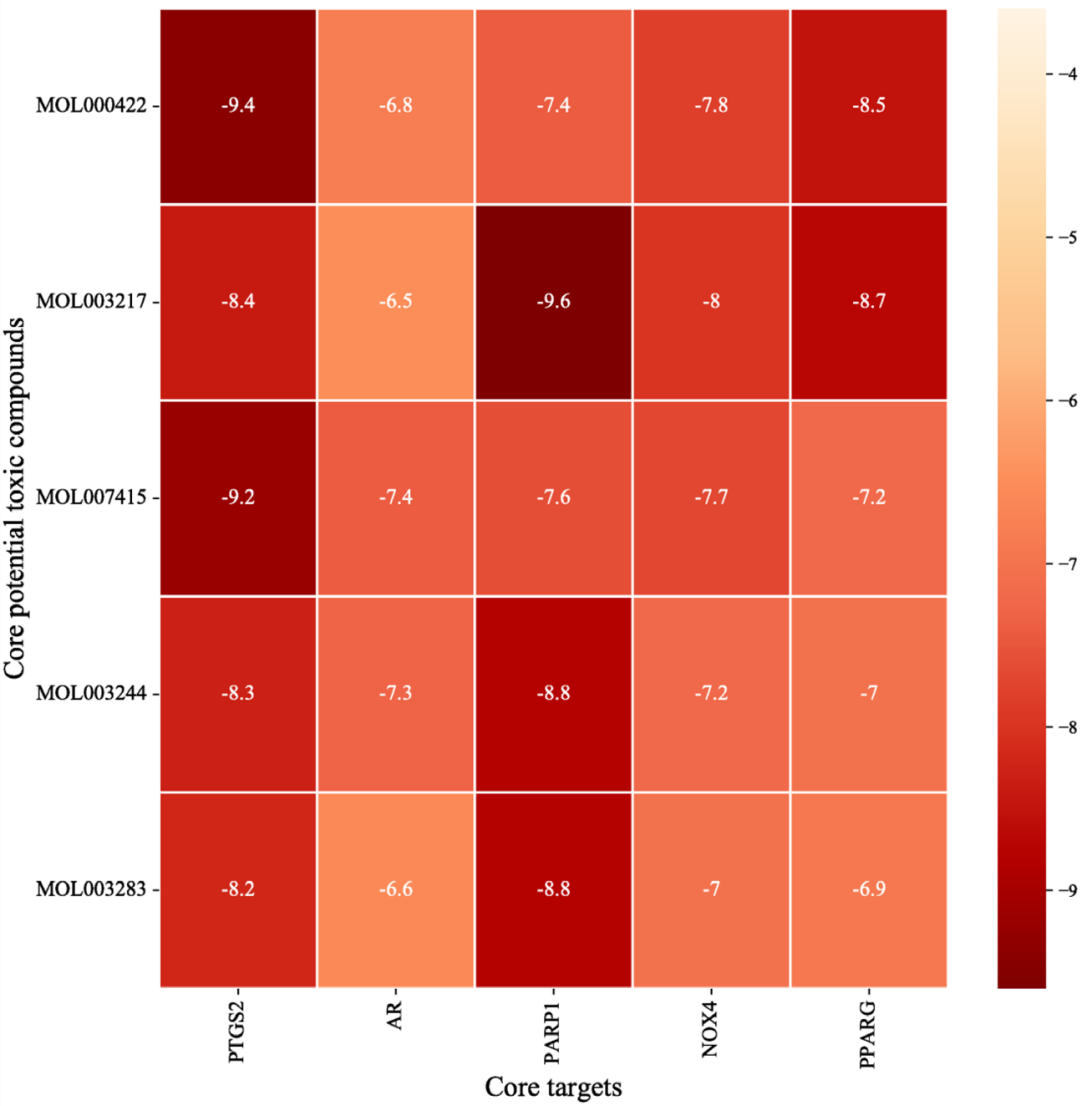
Heatmap of molecular docking Vina score. The darker the color, the smaller the binding energies, the more stable the structure.

**Figure 9.**
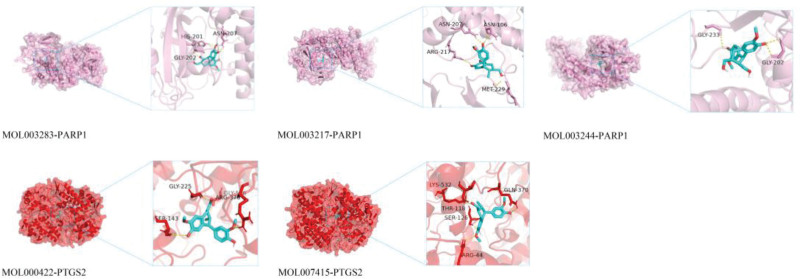
Visualization of top 5 molecular docking Afnity result.

Based on Autodock Vina_1.1.2, we obtained the binding energies (kcal/mol) of core targets and core toxic compounds (Fig. 8). When free binding energies of docking score small than −6.5 kcal/mol, the result was considered to be stable. The heatmap indicated that most docking results were stable and the score of PARP1 docking with Isoxanthohumol, PTGS2 docking with kaempferol, and PTGS2 docking with N-benzoylphenylalanylphenylalinol acetate were more than −9 kcal/mol. To further analysis, we visualized molecular docking results of top 5 high vina scores (Table 3, Fig. 9). The visualization showed that the hydrogen bonds of top 3 high vina scores were 4 which indicated that toxic compounds binding to genes through hydrogen bonds might contribute to toxic effects.

**Table 3:** Molecular docking results of top 5 vina scores.

MOL ID	PubChem ID	Targets	PDB ID	Affinity (kcal/mol)	Box size (x,y,z)	H-bond
MOL003283	160521	PARP1	1UK0	−8.8	(74.94, 89.29, 92.48)	3
MOL003217	513197	PARP1	1UK0	−9.6	(74.94, 89.29, 92.48)	4
MOL003244	65411	PARP1	1UK0	−8.8	(74.94, 89.29, 92.48)	3
MOL000422	5280863	PTGS2	5F19	−9.4	(73.77, 73.77, 101.625)	4
MOL007415	10026486	PTGS2	5F19	−9.2	(73.77, 73.77, 101.625)	4

PARP1 = poly (ADP-Ribose) Polymerase 1, PTGS2 = prostaglandin endoperoxide synthase 2.

## 4. Discussion

When treating some certain diseases, doctors might use some drugs that are harmful to kidneys in order to achieve the purpose of treatment after considering the overall benefit and risk ratio. Therefore, it was important to determine the toxic compounds caused KI that could provide guidance for new TwHF preparations discovery and the subsequent reduction of drug potential KI too. In the study, we found 12 TwHF potential toxic compounds and 62 TwHF induced KI targets. Then we conducted PPI network analysis, gene specific expression analysis, GO function and KEGG pathway enrichment analysis for 62 targets to explore the toxicological mechanism of TwHF. In addition, we selected core toxic compounds and targets for molecular docking to further analysis.

PPI network analysis indicated that TwHF induced KI might be related to hypoxia mediated by HIFIA as well as MTOR^[35,36]^ and inflammation mediated by GSK3B and TNF.^[37,38]^ GO function analysis of cluster 1 from PPI network also confirmed the viewpoint, which was that BP of regulation of inflammatory response and response to oxidative stress were enriched significantly. BioGPS was used to estimate gene expression in organ/tissue/cell, we used it to show the possible sites that may be influenced by TwHF. The results of the study indicated that TwHF induced KI through influencing immune cells and the relationship between KI as well as immune system had been validated.^[39–41]^ In addition, SLC5A2 was the only gene TwHF acted on the kidneys. Solute Carrier Family 5 Member 2 (SLC5A2, also named SGLT2), is responsible for reabsorption of the filtered glucose mostly and associated with familial renal glucosuria.^[42]^ SGLT2 inhibitor could reduce inflammatory and oxidative stress pathways through inducing systematic and glomerular hemodynamic changes.^[43]^ This suggested that TwHF might mediate SLC5A2 leading to renal inflammatory response then to KI, which need further study. GO function enrichment analysis indicated that inflammation and oxidative stress were main process of TwHF induced KI and that was consistent with PPI network analysis and gene expression analysis. KEGG pathway enrichment analysis indicated the signaling transduction pathway of TwHF induced KI. PI3K-Akt signaling pathway was the most important pathway of TwHF induced KI. PI3K-Akt signaling pathway contributed to the development and progression of some inflammatory diseases^[44]^ and Stylianou et al reported that PI3K-Akt pathway was activated in murine lupus nephritis.^[45]^ Liang et al proved that the activation of PI3K-Akt signaling pathway could activate inflammatory response and induce renal structural and functional damages.^[46]^ As for TNF signaling pathway, Tang et al found that TNF signaling pathway was activated in Phospholipase A2 induced AKI,^[47]^ and TNF activation and rising might increased renal tubular cell apoptosis, inflammation and fibrosis in kidneys.^[48,49]^ On the other hand, hypoxia and oxidative stress were common causes to various kidney injury too.^[50]^ HIF1A was a regulator of the cellular response to hypoxia. There was a complex mutual regulation between hypoxia and inflammation,^[51,52]^ and Chen et al validated that tubular epithelial cells exposed to hypoxia would incite tubulointerstitial inflammation.^[53]^ In all, these indicated that TwHF might activate PI3K-Akt signaling pathway as well as TNF signaling pathway to progress renal inflammation, mediate hypoxia via HIF-1 signaling pathway to accelerate inflammatory processes. In addition, AMPK signaling pathway was confirmed to be involved in podocyte injury^[54]^ that provided a probable position of TwHF induced KI, and FoxOs involved in diverse intracellular signaling pathways with critical roles in various physiological as well as pathological conditions^[55]^ while the relationship between FoxOs with KI needed to further research.

Molecular docking results showed that the binding energies of 5 targets and toxic compounds were all less than −6.5 kcal/mol, which verified the binding ability of the targets and compounds screened by network toxicology. In the core potential toxic compounds, Triptonide was the unique compounds in TwHF caused KI. Triptonide, a diterpenoid whose structure was similar to Triptolide, had been confirmed to have nephrotoxicity^[56,57]^ and Ge et al found Triptonide might cause toxic effects through binding to PIK3CA^[58]^ that was correlation with KEGG pathways analysis results. Other core potential toxic compounds such as kaempferol, Isoxanthohumol, N-benzoylphenylalanylphenylalinol acetate with isolariciresinol existed not only in TwHF but also in other TCM. However, renal toxicity had not been reported for them, and researchers needed to pay more attention to them. Triptolide, did not belong to the core toxic compounds as it had zero targets according to the Swiss Target Prediction platform, but there had reports on its renal toxicity.^[59]^ Lu et al found that Triptolide could activate the cGAS-STING signaling pathway within kidney tubular cells in vivo and in vitro, and oxidative stress explained the reason.^[60]^ In addition, Xi et al validated that Triptolide can induce HK-2 cells (Human proximaltubular epithelial cell line) death via apoptotic manners.^[61,62]^ Relatively, ADMET analysis showed that most toxic compounds had high gastrointestinal absorption capacity and low skin permeability, indicating that oral TwHF preparation was more toxic than other dosage forms. The conclusion was also consistent with the TwHF toxicity records from TCM^[63]^ and reminded us that TwHF could be safer when used as external preparations.^[3]^ To be brief, the toxicity of TwHF preparations varied with dosage forms, and it was necessary to continuing determine the toxicity differences of various dosage forms in future studies. In the core targets, PTGS2 and PARP1 demonstrated good docking affinity. PTGS2, also known as cyclooxygenase 2, played a particular role in the inflammatory response^[64]^ and might be involved in ferroptosis, contributing to KI.^[65,66]^ PARP1, which encoded a chromatin-associated enzyme, modified various nuclear proteins through poly (ADP-ribosyl) ation. It was involved in several types of renal diseases and resulted in various degrees of renal proximal tubular epithelial cell injury.^[67,68]^ These mechanisms need to be further studied with animal experiments.

## 5. Conclusion

In the study, we presented a comprehensive investigation into the TwHF induced KI through network toxicology and molecular docking. We determined 12 potential toxic compounds and 62 potential TwHF induced KI targets. Based on PPI network analysis, gene expression analysis and GO function enrichment analysis, we unveiled the key biological processes. We thought the mechanism of TwHF induced KI might associate with inflammation, immune response, hypoxia as well as oxidative stress. Then through KEGG pathway enrichment analysis, we obtained the key signaling pathways such as PI3K-Akt signaling pathway, HIF-1 signaling pathway and TNF signaling pathway. We explored the specific mechanism of TwHF induced KI, which was that TwHF might activate PI3K-Akt signaling pathway as well as TNF signaling pathway to progress renal inflammation, mediate hypoxia via HIF-1 signaling pathway to accelerate inflammatory processes. Finally, we verified the binding ability of the core targets with toxic compounds screened in network toxicology through molecular docking, and this was also helpful for modifying the structure of TwHF toxic compounds as well as reducing TwHF preparations toxicity.

## Acknowledgments

We express gratitude to all authors of the study.

## Author contributions

**Conceptualization:** Yanan Yu, Yanming Xie, Lianxin Wang.

**Data curation:** Zhongming Li.

**Formal analysis:** Shuo Yang, Mengmeng Wang, Xiangjia Luan.

**Funding acquisition:** Lianxin Wang.

**Methodology:** Zhongming Li.

**Supervision:** Junjie Jiang, Yuanyuan Li, Lianxin Wang.

**Visualization:** Shuo Yang, Mengmeng Wang.

**Writing – original draft:** Shuo Yang, Mengmeng Wang.

**Writing – review & editing:** Yanan Yu, Yanming Xie, Lianxin Wang.

## Supplementary Material










