# Supplementary material for: Tripterygium wilfordii Hook.f induced kidney injury through mediating inflammation via PI3K-Akt/HIF-1/TNF signaling pathway: A study of network toxicology and molecular docking
Source: Medicine (Baltimore). 2024 Feb 9;103(6):e36968. doi: 10.1097/MD.0000000000036968 (PMC10860970; doi:10.1097/MD.0000000000036968)
Supplement: Supplementary file 1 [file medi-103-e36968-s001.docx]

Supplemental Table 1 The chemical compounds of TwHF.

|  | **[Mol ID](https://old.tcmsp-e.com/tcmspsearch.php?qr=Tripterygii Radix&qsr=herb_en_name&token=7ff293039932ecfc0a2e2631bc26b05e)** | **[Molecule Name](https://old.tcmsp-e.com/tcmspsearch.php?qr=Tripterygii Radix&qsr=herb_en_name&token=7ff293039932ecfc0a2e2631bc26b05e)** | **[MW](https://old.tcmsp-e.com/tcmspsearch.php?qr=Tripterygii Radix&qsr=herb_en_name&token=7ff293039932ecfc0a2e2631bc26b05e)** | **[OB (%)](https://old.tcmsp-e.com/tcmspsearch.php?qr=Tripterygii Radix&qsr=herb_en_name&token=7ff293039932ecfc0a2e2631bc26b05e)** | **[Caco-2](https://old.tcmsp-e.com/tcmspsearch.php?qr=Tripterygii Radix&qsr=herb_en_name&token=7ff293039932ecfc0a2e2631bc26b05e)** | **[BBB](https://old.tcmsp-e.com/tcmspsearch.php?qr=Tripterygii Radix&qsr=herb_en_name&token=7ff293039932ecfc0a2e2631bc26b05e)** | **[DL](https://old.tcmsp-e.com/tcmspsearch.php?qr=Tripterygii Radix&qsr=herb_en_name&token=7ff293039932ecfc0a2e2631bc26b05e)** |
| --- | --- | --- | --- | --- | --- | --- | --- |
| 1 | MOL000511 | [ursolic acid](https://old.tcmsp-e.com/molecule.php?qn=511) | 456.78 | 16.77 | 0.67 | 0.07 | 0.75 |
| 2 | MOL001452 | [protocatechualdehyde](https://old.tcmsp-e.com/molecule.php?qn=1452) | 138.13 | 38.35 | 0.43 | 0.21 | 0.03 |
| 3 | MOL001739 | [zoomaric acid](https://old.tcmsp-e.com/molecule.php?qn=1739) | 254.46 | 35.78 | 1.18 | 0.88 | 0.1 |
| 4 | MOL001807 | [Cedar acid](https://old.tcmsp-e.com/molecule.php?qn=1807) | 198.19 | 47.78 | 0.5 | 0.1 | 0.06 |
| 5 | MOL001831 | [HX](https://old.tcmsp-e.com/molecule.php?qn=1831) | 136.13 | 52.29 | 0.09 | -0.1 | 0.04 |
| 6 | MOL002249 | [gallocatechin](https://old.tcmsp-e.com/molecule.php?qn=2249) | 306.29 | 2.26 | -0.27 | -1.11 | 0.27 |
| 7 | MOL000295 | [alexandrin](https://old.tcmsp-e.com/molecule.php?qn=295) | 576.95 | 20.63 | -0.2 | -0.81 | 0.63 |
| 8 | MOL000296 | [hederagenin](https://old.tcmsp-e.com/molecule.php?qn=296) | 414.79 | 36.91 | 1.32 | 0.96 | 0.75 |
| 9 | MOL003171 | [2,5-Dimethoxybenzoquinone](https://old.tcmsp-e.com/molecule.php?qn=3171) | 168.16 | 8.72 | 0.47 | -0.04 | 0.04 |
| 10 | MOL003181 | [(2S,3R,4S,5S,6R)-2-[4-[(1S,3aR,4S,6aR)-4-[3-methoxy-4-[(2S,3R,4S,5S,6R)-3,4,5-trihydroxy-6-(hydroxymethyl)oxan-2-yl]oxyphenyl]-1,3,3a,4,6,6a-hexahydrofuro[4,3-c]furan-1-yl]-2,6-dimethoxyphenoxy]-6-(hydroxymethyl)oxane-3,4,5-triol](https://old.tcmsp-e.com/molecule.php?qn=3181) | 712.77 | 9.05 | -2.31 | -3.78 | 0.32 |
| 11 | MOL003182 | [(+)-Medioresinol di-O-beta-D-glucopyranoside_qt](https://old.tcmsp-e.com/molecule.php?qn=3182) | 388.45 | 60.69 | 0.45 | -0.29 | 0.62 |
| 12 | MOL003183 | [TRIPTOTRITERPENIC ACID B](https://old.tcmsp-e.com/molecule.php?qn=3183) | 472.78 | 16.93 | -0.03 | -0.87 | 0.74 |
| 13 | MOL003184 | [81827-74-9](https://old.tcmsp-e.com/molecule.php?qn=3184) | 342.47 | 45.42 | 0.85 | 0.31 | 0.53 |
| 14 | MOL003185 | [(1R,4aR,10aS)-5-hydroxy-1-(hydroxymethyl)-7-isopropyl-8-methoxy-1,4a-dimethyl-4,9,10,10a-tetrahydro-3H-phenanthren-2-one](https://old.tcmsp-e.com/molecule.php?qn=3185) | 346.51 | 48.84 | 0.47 | 0.01 | 0.38 |
| 15 | MOL003186 | [tripterine](https://old.tcmsp-e.com/molecule.php?qn=3186) | 450.67 | 17.84 | 0.46 | -0.34 | 0.78 |
| 16 | MOL003187 | [triptolide](https://old.tcmsp-e.com/molecule.php?qn=3187) | 360.44 | 51.29 | 0.25 | -0.19 | 0.68 |
| 17 | MOL003188 | [Tripchlorolide](https://old.tcmsp-e.com/molecule.php?qn=3188) | 396.9 | 78.72 | 0.16 | -0.31 | 0.72 |
| 18 | MOL003189 | [WILFORLIDE A](https://old.tcmsp-e.com/molecule.php?qn=3189) | 486.81 | 35.66 | 0.31 | -0.57 | 0.72 |
| 19 | MOL003190 | [WILFORLIDE B](https://old.tcmsp-e.com/molecule.php?qn=3190) | 482.77 | 17.67 | 0.47 | -0.13 | 0.56 |
| 20 | MOL003191 | [Tripdiolide](https://old.tcmsp-e.com/molecule.php?qn=3191) | 376.44 | 19.59 | -0.24 | -0.7 | 0.67 |
| 21 | MOL003192 | [Triptonide](https://old.tcmsp-e.com/molecule.php?qn=3192) | 344.39 | 67.66 | 0.15 | -0.29 | 0.7 |
| 22 | MOL003193 | [TRIPTOTRITERPENIC ACID C](https://old.tcmsp-e.com/molecule.php?qn=3193) | 472.78 | 28.91 | 0.2 | -0.53 | 0.74 |
| 23 | MOL003194 | [3-epikatonic acid](https://old.tcmsp-e.com/molecule.php?qn=3194) | 456.78 | 12.47 | 0.57 | -0.1 | 0.76 |
| 24 | MOL003195 | [tripterygone](https://old.tcmsp-e.com/molecule.php?qn=3195) | 454.71 | 14.81 | 0.43 | -0.2 | 0.78 |
| 25 | MOL003196 | [Tryptophenolide](https://old.tcmsp-e.com/molecule.php?qn=3196) | 312.44 | 48.5 | 1.11 | 0.69 | 0.44 |
| 26 | MOL003197 | [orthosphenic acid](https://old.tcmsp-e.com/molecule.php?qn=3197) | 488.78 | 24.14 | -0.17 | -0.81 | 0.6 |
| 27 | MOL003198 | [5 alpha-Benzoyl-4 alpha-hydroxy-1 beta,8 alpha-dinicotinoyl-dihydro-agarofuran](https://old.tcmsp-e.com/molecule.php?qn=3198) | 600.72 | 35.26 | -0.35 | -0.77 | 0.72 |
| 28 | MOL003199 | [5,8-Dihydroxy-7-(4-hydroxy-5-methyl-coumarin-3)-coumarin](https://old.tcmsp-e.com/molecule.php?qn=3199) | 352.31 | 61.85 | 0.02 | -0.71 | 0.54 |
| 29 | MOL003200 | [HRP](https://old.tcmsp-e.com/molecule.php?qn=3200) | 220.25 | 63.93 | -0.03 | -0.41 | 0.1 |
| 30 | MOL003201 | [[(5S,6R,7R,8S)-5,7,8-triacetyloxy-2-[2-(4-methoxyphenyl)ethyl]-4-oxo-5,6,7,8-tetrahydrochromen-6-yl] ethanoate](https://old.tcmsp-e.com/molecule.php?qn=3201) | 516.54 | 28.81 | -0.19 | -0.65 | 0.77 |
| 31 | MOL003202 | [8-epi-Loganic acid](https://old.tcmsp-e.com/molecule.php?qn=3202) | 376.4 | 4.43 | -1.78 | -2.64 | 0.4 |
| 32 | MOL003203 | [8-Epilpganic acid_qt](https://old.tcmsp-e.com/molecule.php?qn=3203) | 214.24 | 98.51 | -0.72 | -1.17 | 0.09 |
| 33 | MOL003204 | [(2S,4S,4aR,6aR,6aS,6bR,8aR,10S,12aR,14bS)-4,10-dihydroxy-2,4a,6a,6b,9,9,12a-heptamethyl-1,3,4,5,6,6a,7,8,8a,10,11,12,13,14b-tetradecahydropicene-2-carboxylic acid](https://old.tcmsp-e.com/molecule.php?qn=3204) | 472.78 | 14.27 | 0.27 | -0.23 | 0.74 |
| 34 | MOL003205 | [Wilforlide A](https://old.tcmsp-e.com/molecule.php?qn=3205) | 454.76 | 14.3 | 0.76 | 0.28 | 0.64 |
| 35 | MOL003206 | [Canin](https://old.tcmsp-e.com/molecule.php?qn=3206) | 278.33 | 77.41 | 0.33 | 0.11 | 0.33 |
| 36 | MOL003207 | [Celacinnine](https://old.tcmsp-e.com/molecule.php?qn=3207) | 405.59 | 28.98 | 0.76 | -0.15 | 0.59 |
| 37 | MOL003208 | [Celafurine](https://old.tcmsp-e.com/molecule.php?qn=3208) | 369.51 | 72.94 | 0.65 | -0.16 | 0.44 |
| 38 | MOL003209 | [Celallocinnine](https://old.tcmsp-e.com/molecule.php?qn=3209) | 405.59 | 83.47 | 0.89 | 0.27 | 0.59 |
| 39 | MOL003210 | [Celapanine](https://old.tcmsp-e.com/molecule.php?qn=3210) | 569.66 | 30.18 | -0.36 | -0.65 | 0.82 |
| 40 | MOL003211 | [Celaxanthin](https://old.tcmsp-e.com/molecule.php?qn=3211) | 550.94 | 47.37 | 1.73 | 0.74 | 0.58 |
| 41 | MOL003212 | [142182-52-3](https://old.tcmsp-e.com/molecule.php?qn=3212) | 342.47 | 19.34 | 0.94 | 0.45 | 0.38 |
| 42 | MOL003213 | [Deoxygomisin A](https://old.tcmsp-e.com/molecule.php?qn=3213) | 400.51 | 20.16 | 1.01 | 0.69 | 0.75 |
| 43 | MOL003214 | [Dunnisinin](https://old.tcmsp-e.com/molecule.php?qn=3214) | 226.25 | 30.68 | -0.1 | -0.41 | 0.13 |
| 44 | MOL003215 | [(2,5-Dioxo-4-imidazolidinyl)carbamic acid](https://old.tcmsp-e.com/molecule.php?qn=3215) | 159.12 | 3.05 | -0.95 | -1.3 | 0.03 |
| 45 | MOL003216 | [trans-Nepetalactone](https://old.tcmsp-e.com/molecule.php?qn=3216) | 166.24 | 70.01 | 1.26 | 1.53 | 0.05 |
| 46 | MOL003217 | [Isoxanthohumol](https://old.tcmsp-e.com/molecule.php?qn=3217) | 354.43 | 56.81 | 0.76 | -0.01 | 0.39 |
| 47 | MOL003218 | [Neouralenol](https://old.tcmsp-e.com/molecule.php?qn=3218) | 370.38 | 12.76 | 0.24 | -0.75 | 0.46 |
| 48 | MOL013351 | [Neoxanthin](https://old.tcmsp-e.com/molecule.php?qn=13351) | 600.96 | 28.01 | 0.42 | -1.61 | 0.51 |
| 49 | MOL003220 | [[(3aR,4S,6E,9S,10Z,11aR)-9-hydroxy-6,10-dimethyl-3-methylene-2-oxo-3a,4,5,8,9,11a-hexahydrocyclodeca[b]furan-4-yl] (E)-2-methylbut-2-enoate](https://old.tcmsp-e.com/molecule.php?qn=3220) | 346.46 | 12.31 | 0.37 | 0.11 | 0.31 |
| 50 | MOL003221 | [Regelindiol A](https://old.tcmsp-e.com/molecule.php?qn=3221) | 486.81 | 17.68 | 0.44 | -0.26 | 0.72 |
| 51 | MOL003222 | [Salazinic acid](https://old.tcmsp-e.com/molecule.php?qn=3222) | 402.33 | 36.34 | -1.2 | -1.81 | 0.76 |
| 52 | MOL003223 | [Tingenone](https://old.tcmsp-e.com/molecule.php?qn=3223) | 420.64 | 12.04 | 0.67 | 0.03 | 0.8 |
| 53 | MOL003224 | [Tripdiotolnide](https://old.tcmsp-e.com/molecule.php?qn=3224) | 360.44 | 56.4 | -0.29 | -0.65 | 0.67 |
| 54 | MOL003225 | [Hypodiolide A](https://old.tcmsp-e.com/molecule.php?qn=3225) | 318.5 | 76.13 | 0.65 | 0.28 | 0.49 |
| 55 | MOL003226 | [(24Z)-27-Hydroxy-3-oxo-7,24-tirucalladien-21-al](https://old.tcmsp-e.com/molecule.php?qn=3226) | 454.76 | 25.4 | 0.29 | -0.65 | 0.82 |
| 56 | MOL003227 | [Tripteroside](https://old.tcmsp-e.com/molecule.php?qn=3227) | 422.37 | 2.74 | -1.36 | -2.38 | 0.78 |
| 57 | MOL003228 | [Norathyriol](https://old.tcmsp-e.com/molecule.php?qn=3228) | 260.21 | 18.35 | 0.15 | -0.73 | 0.22 |
| 58 | MOL003229 | [Triptinin B](https://old.tcmsp-e.com/molecule.php?qn=3229) | 314.46 | 34.73 | 0.84 | 0.38 | 0.32 |
| 59 | MOL003230 | [Triptodihydroxy acid methyl ester](https://old.tcmsp-e.com/molecule.php?qn=3230) | 486.81 | 15.76 | 0.3 | -0.59 | 0.73 |
| 60 | MOL003231 | [Triptoditerpenic acid B](https://old.tcmsp-e.com/molecule.php?qn=3231) | 328.49 | 40.02 | 0.97 | 0.7 | 0.36 |
| 61 | MOL003232 | [Triptofordin B1](https://old.tcmsp-e.com/molecule.php?qn=3232) | 478.63 | 39.55 | 0.41 | -0.16 | 0.84 |
| 62 | MOL003233 | [Triptofordin B2](https://old.tcmsp-e.com/molecule.php?qn=3233) | 608.69 | 107.71 | -0.65 | -0.96 | 0.76 |
| 63 | MOL003234 | [Triptofordin C2](https://old.tcmsp-e.com/molecule.php?qn=3234) | 610.71 | 30.16 | -0.65 | -1.09 | 0.76 |
| 64 | MOL003235 | [Triptofordin D1](https://old.tcmsp-e.com/molecule.php?qn=3235) | 606.72 | 32 | -0.35 | -0.61 | 0.75 |
| 65 | MOL003236 | [Triptofordin D2](https://old.tcmsp-e.com/molecule.php?qn=3236) | 650.78 | 30.38 | -0.47 | -0.75 | 0.69 |
| 66 | MOL003237 | [(3E,7E)-2alpha,10beta,13alpha-Triacetoxy-5alpha,20-dihydroxy-3,8-seco-taxa-3,7,11-trien-9-one](https://old.tcmsp-e.com/molecule.php?qn=3237) | 492.62 | 7.93 | -0.81 | -1.38 | 0.69 |
| 67 | MOL003238 | [Triptofordin F1](https://old.tcmsp-e.com/molecule.php?qn=3238) | 694.79 | 33.91 | -0.96 | -1.24 | 0.6 |
| 68 | MOL003239 | [Triptofordin F2](https://old.tcmsp-e.com/molecule.php?qn=3239) | 668.75 | 33.62 | -0.98 | -1.32 | 0.67 |
| 69 | MOL003240 | [Triptofordin F3](https://old.tcmsp-e.com/molecule.php?qn=3240) | 710.79 | 8.04 | -0.45 | -1.1 | 0.6 |
| 70 | MOL003241 | [Triptofordin F4](https://old.tcmsp-e.com/molecule.php?qn=3241) | 652.75 | 31.37 | -0.87 | -1.05 | 0.67 |
| 71 | MOL003242 | [Triptofordinine A2](https://old.tcmsp-e.com/molecule.php?qn=3242) | 741.85 | 30.78 | -0.92 | -0.99 | 0.47 |
| 72 | MOL003243 | [Triptolidenol](https://old.tcmsp-e.com/molecule.php?qn=3243) | 376.44 | 18.52 | -0.08 | -0.53 | 0.66 |
| 73 | MOL003244 | [Triptonide](https://old.tcmsp-e.com/molecule.php?qn=3244) | 358.42 | 68.45 | 0.15 | -0.3 | 0.68 |
| 74 | MOL003245 | [Triptonoditerpenic acid](https://old.tcmsp-e.com/molecule.php?qn=3245) | 344.49 | 42.56 | 0.81 | 0.27 | 0.39 |
| 75 | MOL003246 | [11-Hydroxy-14,15alpha-epoxytabersonine](https://old.tcmsp-e.com/molecule.php?qn=3246) | 368.47 | 13.47 | 0.45 | 0.02 | 0.81 |
| 76 | MOL003247 | [Triptonoterpene methyl ether](https://old.tcmsp-e.com/molecule.php?qn=3247) | 330.51 | 16.46 | 0.97 | 0.59 | 0.34 |
| 77 | MOL003248 | [Triptonoterpene](https://old.tcmsp-e.com/molecule.php?qn=3248) | 300.48 | 48.57 | 1.22 | 0.96 | 0.28 |
| 78 | MOL003249 | [Triptoriterpenic acid A](https://old.tcmsp-e.com/molecule.php?qn=3249) | 488.78 | 9.69 | 0.02 | -0.61 | 0.71 |
| 79 | MOL003250 | [Triptotin](https://old.tcmsp-e.com/molecule.php?qn=3250) | 472.78 | 17.66 | 0.43 | -0.16 | 0.61 |
| 80 | MOL003251 | [Triptotriterpenic acid A](https://old.tcmsp-e.com/molecule.php?qn=3251) | 472.78 | 17.2 | -0.06 | -0.78 | 0.74 |
| 81 | MOL003252 | [Triptotriterpenic acid B](https://old.tcmsp-e.com/molecule.php?qn=3252) | 472.78 | 13.09 | 0.14 | -0.59 | 0.74 |
| 82 | MOL003253 | [Triptotriterpenic acid C](https://old.tcmsp-e.com/molecule.php?qn=3253) | 472.78 | 11.85 | -0.04 | -0.75 | 0.74 |
| 83 | MOL003254 | [Triptotriterpenoidel lactone A](https://old.tcmsp-e.com/molecule.php?qn=3254) | 452.79 | 19.13 | 0.84 | 0.2 | 0.63 |
| 84 | MOL003255 | [WILFORNINE A](https://old.tcmsp-e.com/molecule.php?qn=3255) | 925.97 | 103 | -1.25 | -2 | 0.13 |
| 85 | MOL003256 | [WILFORNINE B](https://old.tcmsp-e.com/molecule.php?qn=3256) | 883.93 | 25.86 | -1.25 | -1.77 | 0.15 |
| 86 | MOL003257 | [WILFORNINE C](https://old.tcmsp-e.com/molecule.php?qn=3257) | 988.04 | 112.69 | -1.27 | -1.79 | 0.1 |
| 87 | MOL003258 | [WILFORNINE D](https://old.tcmsp-e.com/molecule.php?qn=3258) | 915.93 | 24.39 | -1.12 | -1.65 | 0.14 |
| 88 | MOL003259 | [WILFORNINE E](https://old.tcmsp-e.com/molecule.php?qn=3259) | 777.8 | 17.74 | -1.57 | -1.71 | 0.22 |
| 89 | MOL003260 | [WILFORNINE F](https://old.tcmsp-e.com/molecule.php?qn=3260) | 825.89 | 51.71 | -0.67 | -0.97 | 0.17 |
| 90 | MOL003261 | [WILFORNINE I](https://old.tcmsp-e.com/molecule.php?qn=3261) | 946 | 27.67 | -1.17 | -1.58 | 0.11 |
| 91 | MOL003262 | [WILFORNINE J](https://old.tcmsp-e.com/molecule.php?qn=3262) | 763.82 | 17.7 | -1.16 | -1.4 | 0.25 |
| 92 | MOL003263 | [Wilfordic acid](https://old.tcmsp-e.com/molecule.php?qn=3263) | 223.25 | 33.99 | 0.05 | -0.22 | 0.08 |
| 93 | MOL003264 | [Wilfordside](https://old.tcmsp-e.com/molecule.php?qn=3264) | 883.93 | 29.81 | -1 | -1.4 | 0.15 |
| 94 | MOL003265 | [Wilforlide B](https://old.tcmsp-e.com/molecule.php?qn=3265) | 452.74 | 24.77 | 0.85 | 0.45 | 0.64 |
| 95 | MOL003266 | [21-Hydroxy-30-norhopan-22-one](https://old.tcmsp-e.com/molecule.php?qn=3266) | 428.77 | 34.11 | 0.9 | 0.54 | 0.77 |
| 96 | MOL003267 | [Wilformine](https://old.tcmsp-e.com/molecule.php?qn=3267) | 805.86 | 46.32 | -1.1 | -1.33 | 0.2 |
| 97 | MOL003268 | [Wilfornine](https://old.tcmsp-e.com/molecule.php?qn=3268) | 878.96 | 102.49 | -0.99 | -1.69 | 0.1 |
| 98 | MOL003269 | [104331-87-5](https://old.tcmsp-e.com/molecule.php?qn=3269) | 220.29 | 54.85 | 0.53 | 0.2 | 0.13 |
| 99 | MOL003270 | [Wilforzine](https://old.tcmsp-e.com/molecule.php?qn=3270) | 825.89 | 49.33 | -1 | -1.68 | 0.16 |
| 100 | MOL003271 | [Wilfotrine](https://old.tcmsp-e.com/molecule.php?qn=3271) | 873.89 | 43.32 | -1.44 | -1.69 | 0.15 |
| 101 | MOL003272 | [Wilsonine](https://old.tcmsp-e.com/molecule.php?qn=3272) | 343.46 | 29.27 | 1.09 | 0.84 | 0.65 |
| 102 | MOL003273 | [euonine](https://old.tcmsp-e.com/molecule.php?qn=3273) | 805.86 | 7.5 | -1.6 | -1.94 | 0.2 |
| 103 | MOL003274 | [neowilforine](https://old.tcmsp-e.com/molecule.php?qn=3274) | 849.96 | 67.15 | -0.6 | -1.06 | 0.15 |
| 104 | MOL003275 | [wilfordine](https://old.tcmsp-e.com/molecule.php?qn=3275) | 883.93 | 7.5 | -1.23 | -1.52 | 0.14 |
| 105 | MOL003276 | [wilforine](https://old.tcmsp-e.com/molecule.php?qn=3276) | 867.93 | 7.51 | -0.82 | -1.34 | 0.15 |
| 106 | MOL003277 | [wilfortrine](https://old.tcmsp-e.com/molecule.php?qn=3277) | 873.89 | 43.57 | -1.51 | -1.82 | 0.15 |
| 107 | MOL003278 | [salaspermic acid](https://old.tcmsp-e.com/molecule.php?qn=3278) | 472.78 | 32.19 | 0.3 | -0.4 | 0.63 |
| 108 | MOL003279 | [99694-86-7](https://old.tcmsp-e.com/molecule.php?qn=3279) | 376.44 | 75.23 | -0.13 | -0.55 | 0.66 |
| 109 | MOL003280 | [TRIPTONOLIDE](https://old.tcmsp-e.com/molecule.php?qn=3280) | 326.42 | 49.51 | 0.72 | 0.1 | 0.49 |
| 110 | MOL000346 | [succinic acid](https://old.tcmsp-e.com/molecule.php?qn=346) | 118.1 | 29.62 | -0.44 | -0.71 | 0.01 |
| 111 | MOL000358 | [beta-sitosterol](https://old.tcmsp-e.com/molecule.php?qn=358) | 414.79 | 36.91 | 1.32 | 0.99 | 0.75 |
| 112 | MOL000365 | [syringaresinol](https://old.tcmsp-e.com/molecule.php?qn=365) | 418.48 | 3.29 | 0.6 | -0.03 | 0.72 |
| 113 | MOL000616 | [(+)-Suyringaresinol-di-O-beta-D-glucoside](https://old.tcmsp-e.com/molecule.php?qn=616) | 742.8 | 5.19 | -2.14 | -3.49 | 0.29 |
| 114 | MOL000676 | [DBP](https://old.tcmsp-e.com/molecule.php?qn=676) | 278.38 | 64.54 | 0.8 | 0.56 | 0.13 |
| 115 | MOL000057 | [DIBP](https://old.tcmsp-e.com/molecule.php?qn=57) | 278.38 | 49.63 | 0.85 | 0.68 | 0.13 |
| 116 | MOL000069 | [palmitic acid](https://old.tcmsp-e.com/molecule.php?qn=69) | 256.48 | 19.3 | 1.09 | 1 | 0.1 |
| 117 | MOL000211 | [Mairin](https://old.tcmsp-e.com/molecule.php?qn=211) | 456.78 | 55.38 | 0.73 | 0.22 | 0.78 |
| 118 | MOL000263 | [oleanolic acid](https://old.tcmsp-e.com/molecule.php?qn=263) | 456.78 | 29.02 | 0.59 | 0.07 | 0.76 |
| 119 | MOL000357 | [Sitogluside](https://old.tcmsp-e.com/molecule.php?qn=357) | 576.95 | 20.63 | -0.14 | -0.93 | 0.62 |
| 120 | MOL000361 | [Amyrin](https://old.tcmsp-e.com/molecule.php?qn=361) | 426.8 | 17.6 | 1.45 | 1.29 | 0.76 |
| 121 | MOL000396 | [(+)-Syringaresinol](https://old.tcmsp-e.com/molecule.php?qn=396) | 418.48 | 3.29 | 0.47 | -0.34 | 0.72 |
| 122 | MOL000422 | [kaempferol](https://old.tcmsp-e.com/molecule.php?qn=422) | 286.25 | 41.88 | 0.26 | -0.55 | 0.24 |
| 123 | MOL000449 | [Stigmasterol](https://old.tcmsp-e.com/molecule.php?qn=449) | 412.77 | 43.83 | 1.44 | 1 | 0.76 |
| 124 | MOL000508 | [Friedelin](https://old.tcmsp-e.com/molecule.php?qn=508) | 426.8 | 29.16 | 1.43 | 1.38 | 0.76 |
| 125 | MOL000550 | [meso-galactitol](https://old.tcmsp-e.com/molecule.php?qn=550) | 182.2 | 10.69 | -1.84 | -4.33 | 0.03 |
| 126 | MOL000860 | [stearic acid](https://old.tcmsp-e.com/molecule.php?qn=860) | 284.54 | 17.83 | 1.15 | 1.22 | 0.14 |
| 127 | MOL000879 | [methyl palmitate](https://old.tcmsp-e.com/molecule.php?qn=879) | 270.51 | 18.09 | 1.37 | 1.18 | 0.12 |
| 128 | MOL002001 | [Oleanolic acid deriv.](https://old.tcmsp-e.com/molecule.php?qn=2001) | 498.82 | 14.24 | 0.65 | 0.13 | 0.7 |
| 129 | MOL002058 | [40957-99-1](https://old.tcmsp-e.com/molecule.php?qn=2058) | 388.45 | 57.2 | 0.49 | -0.29 | 0.62 |
| 130 | MOL003283 | [(2R,3R,4S)-4-(4-hydroxy-3-methoxy-phenyl)-7-methoxy-2,3-dimethylol-tetralin-6-ol](https://old.tcmsp-e.com/molecule.php?qn=3283) | 360.44 | 66.51 | -0.2 | -1.17 | 0.39 |
| 131 | MOL003973 | [caffeine](https://old.tcmsp-e.com/molecule.php?qn=3973) | 194.22 | 89.46 | 0.58 | -0.01 | 0.08 |
| 132 | MOL004175 | [NSC733507](https://old.tcmsp-e.com/molecule.php?qn=4175) | 488.78 | 17.32 | -0.22 | -0.8 | 0.71 |
| 133 | MOL004443 | [Zhebeiresinol](https://old.tcmsp-e.com/molecule.php?qn=4443) | 280.3 | 58.72 | 0.53 | 0.06 | 0.19 |
| 134 | MOL004667 | [fraxetin](https://old.tcmsp-e.com/molecule.php?qn=4667) | 208.18 | 23.04 | 0.51 | 0.36 | 0.09 |
| 135 | MOL005828 | [nobiletin](https://old.tcmsp-e.com/molecule.php?qn=5828) | 402.43 | 61.67 | 1.05 | -0.08 | 0.52 |
| 136 | MOL006364 | [(2R,3R,4S)-4-(4-hydroxy-3,5-dimethoxy-phenyl)-5,7-dimethoxy-2,3-dimethylol-tetralin-6-ol](https://old.tcmsp-e.com/molecule.php?qn=6364) | 420.5 | 4.87 | -0.16 | -0.91 | 0.54 |
| 137 | MOL006384 | [4-[(1R,3aS,4R,6aS)-4-(4-hydroxy-3,5-dimethoxyphenyl)-1,3,3a,4,6,6a-hexahydrofuro[4,3-c]furan-1-yl]-2,6-dimethoxyphenol](https://old.tcmsp-e.com/molecule.php?qn=6384) | 418.48 | 3.29 | 0.66 | -0.39 | 0.72 |
| 138 | MOL006791 | [epigallocatechin](https://old.tcmsp-e.com/molecule.php?qn=6791) | 306.29 | 24.18 | -0.22 | -0.82 | 0.27 |
| 139 | MOL007415 | [[(2S)-2-[[(2S)-2-(benzoylamino)-3-phenylpropanoyl]amino]-3-phenylpropyl] acetate](https://old.tcmsp-e.com/molecule.php?qn=7415) | 444.57 | 58.02 | 0.32 | -0.26 | 0.52 |
| 140 | MOL007535 | [(5S,8S,9S,10R,13R,14S,17R)-17-[(1R,4R)-4-ethyl-1,5-dimethylhexyl]-10,13-dimethyl-2,4,5,7,8,9,11,12,14,15,16,17-dodecahydro-1H-cyclopenta[a]phenanthrene-3,6-dione](https://old.tcmsp-e.com/molecule.php?qn=7535) | 428.77 | 33.12 | 0.9 | 0.44 | 0.79 |
| 141 | MOL007569 | [3-hydroxy-1-(3,5-dimethoxy-4-hydroxyphenyl)propan-1-one](https://old.tcmsp-e.com/molecule.php?qn=7569) | 226.25 | 9.91 | 0.13 | -0.57 | 0.08 |
| 142 | MOL009386 | [3,3'-bis-(3,4-dihydro-4-hydroxy-6-methoxy)-2H-1-benzopyran](https://old.tcmsp-e.com/molecule.php?qn=9386) | 358.42 | 52.11 | 0.14 | -0.55 | 0.54 |
| 143 | MOL011169 | [Peroxyergosterol](https://old.tcmsp-e.com/molecule.php?qn=11169) | 428.72 | 44.39 | 0.86 | 0.43 | 0.82 |
| 144 | MOL012680 | [Antiarol](https://old.tcmsp-e.com/molecule.php?qn=12680) | 184.21 | 71.24 | 1.02 | 0.78 | 0.05 |
