# Supplementary material for: Tripterygium wilfordii Hook.f induced kidney injury through mediating inflammation via PI3K-Akt/HIF-1/TNF signaling pathway: A study of network toxicology and molecular docking
Source: Medicine (Baltimore). 2024 Feb 9;103(6):e36968. doi: 10.1097/MD.0000000000036968 (PMC10860970; doi:10.1097/MD.0000000000036968)
Supplement: Supplementary file 2 [file medi-103-e36968-s002.docx]

Supplemental Table 2 TwHF targets.

NPC1L1

NR1H3

RORC

SHBG

HMGCR

CYP17A1

SREBF2

CYP19A1

AR

CYP51A1

RORA

ESR1

ESR2

PTPN1

CYP2C19

ACHE

SERPINA6

G6PD

SLC6A2

SLC6A4

BCHE

CHRM2

NR1I3

NR1H2

VDR

PTGER1

PTGER2

TBXAS1

PTGES

DHCR7

PPARD

SQLE

GLRA1

HSD11B1

PTPN6

PTPN2

FDFT1

CES2

NOS2

PPARG

UGT2B7

NR3C1

POLB

NOX4

AKR1B1

XDH

TYR

FLT3

CA2

ALOX5

CA7

HSD17B2

ABCC1

HSD17B1

AHR

CA12

ESRRA

ABCB1

CYP1B1

ABCG2

ADORA1

CA4

MAOA

GLO1

SYK

GSK3B

MMP9

MMP2

ALOX15

ALOX12

PTPRS

ADORA2A

CDK5R1

CCNB3

ARG1

GPR35

DAPK1

MPG

SLC22A12

TTR

AKR1B10

TNKS2

TNKS

CDK6

CDK2

CSNK2A1

EGFR

AVPR2

IGF1R

F2

PIM1

AURKB

DRD4

MPO

PIK3R1

PYGL

CA1

SRC

PTK2

KDR

MMP13

MMP3

CA3

PLK1

CA6

CDK1

PKN1

CA14

CA9

MET

NEK2

CXCR1

CAMK2B

ALK

AKT1

NEK6

PLA2G1B

CA5A

BACE1

AXL

NUAK1

AKR1C2

AKR1C1

AKR1C3

AKR1C4

CA13

AKR1A1

APP

PARP1

MMP12

CD38

TOP1

PTGS2

CFTR

PFKFB3

AMY1A

GRK6

TERT

MAPT

CCNB1

CCNB2

CDK5

PPARA

NEK1

DRD2

PLA2G7

HTR2A

PRKCZ

SRD5A2

PTGS1

SLC5A2

CES1

RXRA

ADORA3

TAS2R31

MAOB

CTSL

CTSK

ODC1

PLA2G2A

PLA2G5

PLA2G10

KLK2

CBR1

ADAM17

MAPK8

CLK1

DYRK1B

NR1H4

MMP1

MMP8

DUT

HSP90AA1

BRAF

OPRM1

OPRD1

RPS6KB1

AURKA

MDM4

CSF1R

SGK1

MDM2

PDK1

EDNRA

MMP7

CCNE1

TNF

CFD

CTSD

PDE6C

MMP25

MMP16

BMP1

GRK2

ADAM10

CCND1

RET

CCNE2

AGTR1

LCK

SIRT3

SIRT1

CSNK1G1

RPS6KA1

ROCK1

MAPK1

TNNI3K

CAMK2D

DPP4

DPP7

DPP8

TAAR1

CDK3

CDK4

F2RL1

KCNA3

NR3C2

GSTM1

ATP2A1

PCSK7

P2RX3

CASP8

CASP1

ADRA1D

PGR

ERBB2

MAPK10

VAV1

CAPN1

SCN9A

PPP2CA

ABCC9

TOP2A

NFKBIA

RELA

IARS

OPRK1

MAPK14

ADORA2B

SLC5A1

EWS-Fli1

BDKRB1

IDO1

HSD11B2

KDM5C

KDM4B

KDM5B

KDM4A

HDAC6

HDAC1

KIF11

EDNRB

PDE10A

POLA1

CYP24A1

WEE1

MKNK2

CHEK1

ACVRL1

ERN1

MAP2K2

MAPKAPK5

TYK2

PLK4

MAP3K14

ADK

HTR1A

NTRK1

ADCY10

PIK3CA

MAP3K7

MTOR

ESRRB

GPER1

TYRO3

MERTK

JUN

ITK

MAP2K7

KIT

CCR3

CTSB

TACR2

ACE

CTSS

F10

HLA-DRB1

PSMB5

CBX7

ITGA2B

PSENEN

GHSR

CTSV

IDH1

SOAT1

CCKBR

FLT1

FGFR1

GABRB3

GABRA2

GABRA1

CMA1

S1PR3

PDPK1

CTSG

DYRK1A

TBK1

CDK8

CACNA1C

FNTA

PDGFRA

TBXA2R

PDE2A

HDAC2

PDE4D

PDE4C

ALOX5AP

PAM

TGM1

KCNJ5

MAP2K1

F13A1

RHOA

CBX8

CBX4

SLC7A11

METAP1

GPR183

VDAC2

LRRK2

PDGFRB

MAPKAPK2

RPS6KA3

MAPK11

HIF1A

NAMPT

TSHR

JAK2

PRKDC

NTSR1

CETP

PABPC1

SLC27A1

JAK3

TGFBR1

TACR1

KCNJ3

FNTB

GABRA3

GABRB2

ITGB3

GABRG2

CNR1

CNR2

PDE4A

PDE4B

SHH

HPGDS

TRPV1

FAAH

FASN
