# Supplementary material for: Tripterygium wilfordii Hook.f induced kidney injury through mediating inflammation via PI3K-Akt/HIF-1/TNF signaling pathway: A study of network toxicology and molecular docking
Source: Medicine (Baltimore). 2024 Feb 9;103(6):e36968. doi: 10.1097/MD.0000000000036968 (PMC10860970; doi:10.1097/MD.0000000000036968)
Supplement: Supplementary file 3 [file medi-103-e36968-s003.docx]

Supplemental Table 3 Kidney injury relevant targets.

|  | **GeneCards Database** | **OMIM Database** | **DisGeNET Database** |
| --- | --- | --- | --- |
| EDNRB | LCN2 | CLCNKA | HAVCR1 |
| TP53BP1 | HAVCR1 | CLCNKB | CLU |
| BMPR2 | MGAT3-AS1 | ALPL | LCN2 |
|  | CFH | MUC1 | NPPA |
|  | CST3 | COPA | PPARG |
|  | SLC22A12 | PBX1 | B2M |
|  | SLC2A9 | REN | EGFR |
|  | IL18 | DSTYK | NOS3 |
|  | ADAMTS13 | SEC61A1 | MPO |
|  | DGKE | DZIP1L | NFE2L2 |
|  | TNF | DNAJB11 | BAX |
|  | IL6 | PKD2 | HMOX1 |
|  | B2M | KAAG1 | NQO1 |
|  | ACE | SLC17A1 | HBEGF |
|  | THBD | HLA-B | HP |
|  | ALB | FCYT | TLR4 |
|  | CFI | TBX18 | GFER |
|  | C3 | CRB2 | ATP5F1B |
|  | CD46 | GANAB | SIRT1 |
|  | TP53 | LRP5 | C3 |
|  | H19 | ALG8 | SLC22A12 |
|  | SOD2-OT1 | ZPR1 | ALB |
|  | CFHR1 | NINJ2 | G6PD |
|  | PVT1 | AQP6 | TNF |
|  | CFHR3 | ALG5 | SLC22A24 |
|  | PGR-AS1 | SLC25A30 | HBG2 |
|  | MB | RHCG | HSPA1A |
|  | TIMP2 | PKDTS | SPP1 |
|  | MALAT1 | PKD1 | VEGFA |
|  | HPRT1 | UMOD | EPO |
|  | UMOD | NRIP1 | AMBP |
|  | IGFBP7 | PKDREJ | KNG1 |
|  | MEG3 | ADTKD1 | CST3 |
|  | MIR21 | RIP140 | GSTP1 |
|  | FABP1 | HOPS | NOS2 |
|  | APRT | PUM | ORM1 |
|  | RYR1 | AILJK | HPX |
|  | NPPA | CAKUHED | NFKB1 |
|  | GPT | RTD | HEXB |
|  | HP | KIAA0472 | MTHFR |
|  | NOS3 | SEC61 | IL6 |
|  | CRP | DZIP2 | MIR215 |
|  | PIK3C2A | HEDJ | MIR192 |
|  | AMBP | RU2AS | UOX |
|  | REN | NPT1 | MIR148A |
|  | GAS5 | SPDA1 | LRP2 |
|  | LINC01672 | PKHD1 | MIRLET7I |
|  | MIR494 | CAKUT2 | MIR10A |
|  | FGF23 | FSGS9 | MIR122 |
|  | IL10 | PKD3 | MIR140 |
|  | CERNA3 | BMND1 | KLK1 |
|  | MIR125A | CDG1H | MIR23A |
|  | COL4A5 | ZNF259 | MIR99A |
|  | AGT | AQP2L | MIR30D |
|  | CFB | PKD7 | IFNA2 |
|  | SLC9A3 | KMCP1 | IGF1 |
|  | CFHR5 | RHGK | MIR30A |
|  | F2 | RIP5 | IGFBP1 |
|  | RBP4 | DUSTYPK | MIR25 |
|  | EPO | CAKUT1 | IL10 |
|  | HNF1B | SPG23 | INS |
|  | MIR15A | DJ9 | MIR143 |
|  | NPHS2 | ABBP2 | GSTM2 |
|  | NPPB | PKD6 | GSTA1 |
|  | ICAM1 | PKD5 | TF |
|  | CCAT1 | ADTKD5 | TFF3 |
|  | KNG1 | ADTKD2 | CD44 |
|  | HMOX1 | TNSALP | CYP2D6 |
|  | LDHA | HPPA | CYP2C9 |
|  | LPIN1 | HPPO | CYP2C19 |
|  | MIRLET7B | HPPI | TGFB1 |
|  | PKD2 | HPPC | THBD |
|  | NAGLU | ADTKD4 | CP |
|  | HGF | LRP7 | CCR5 |
|  | TCN2 | LR3 | TLR2 |
|  | ENSG00000275307 | OPPG | FSTL1 |
|  | ENSG00000276965 | OPTA1 | TP53 |
|  | MIR146B | EVR4 | TTR |
|  | CFHR2 | PCLD4 | GPNMB |
|  | MYD88 | CAKUT3 | RAPGEF3 |
|  | PYGM | ARPKD | AMN |
|  | TRA-TGC7-1 | PKD4 | AFM |
|  | TRA-TGC5-1 | VMCKD | SOD1 |
|  | TLR4 | PCLD3 | EDN1 |
|  | YAP1 | GKAF | GSK3B |
|  | CXCL8 |  | IL20 |
|  | FLT1 |  | TNFRSF12A |
|  | HAVCR2 |  | GC |
|  | CACNA1S |  | SERPINA1 |
|  | CRBN |  | GAS6 |
|  | NOS2 |  | PLG |
|  | HSPA1A |  | POMC |
|  | PKD1 |  | UGT1A9 |
|  | MIR93 |  | UGT1A1 |
|  | CPT2 |  | AVP |
|  | SLC22A6 |  | MIR146B |
|  | MIR106B |  | EPHX2 |
|  | TUG1 |  | BDKRB2 |
|  | XIST |  | A2M |
|  | PRPS1 |  | AHSG |
|  | PIGA |  | SLC11A1 |
|  | IFT140 |  | OCLN |
|  | PKHD1 |  | HIF1A |
|  | MT-CO1 |  | FN1 |
|  | LINC02605 |  | HES1 |
|  | CCL2 |  | CYP2E1 |
|  | SIRT1 |  | ADRB2 |
|  | CXCL10 |  | XDH |
|  | APOL1 |  | IL1B |
|  | MMACHC |  | ECE1 |
|  | NLRP3 |  | SLC22A2 |
|  | ASPH |  | BCL2 |
|  | HOTAIR |  | RB1 |
|  | G6PD |  | BCL2L1 |
|  | CD36 |  | ALAD |
|  | NTN1 |  | PTGS2 |
|  | TMX2-CTNND1 |  | PTGER4 |
|  | CFHR4 |  | PTAFR |
|  | COMT |  | BAD |
|  | BDNF-AS |  | FGA |
|  | HLA-A |  | MFN1 |
|  | HAMP |  | AVPR1A |
|  | ANGPT2 |  | EPAS1 |
|  | KL |  | EGF |
|  | IL7R |  | SLC22A1 |
|  | ACE2 |  | CASP1 |
|  | EGF |  | CAPN1 |
|  | TGFB1 |  | CDKN1B |
|  | APOE |  | PROCR |
|  | EDN1 |  | NES |
|  | ENSG00000277577 |  | EGLN3 |
|  | HIF1A |  | CRP |
|  | SFTA3 |  | TGFA |
|  | AGTR1 |  | CYP27B1 |
|  | LGALS3 |  | ACE |
|  | PRTN3 |  | DDIT3 |
|  | HLA-B |  | HDAC3 |
|  | CR1 |  | MTOR |
|  | IL17A |  | AR |
|  | CCND1 |  | ICAM1 |
|  | ALDOA |  | SCGB1A1 |
|  | STAT2 |  | NPPB |
|  | CORIN |  | MIR26A1 |
|  | CLCNKB |  | NOS1 |
|  | CLCNKA |  | IGF1R |
|  | BSND |  | NDUFB8 |
|  | SLC26A1 |  | ARG2 |
|  | SAA1 |  | HSPA8 |
|  | STOX1 |  | MET |
|  | DZIP1L |  | MB |
|  | MT-CO3 |  | ANXA1 |
|  | HELLPAR |  | GSR |
|  | IGF1 |  | UTS2R |
|  | TNFRSF1A |  | HDAC5 |
|  | MIF |  | EGLN1 |
|  | MST1R |  | ALOX5 |
|  | VCAM1 |  | WNT4 |
|  | TTR |  | LGALS3 |
|  | FLT3 |  | PKD2 |
|  | LCAT |  | GATM |
|  | HBB |  | FIS1 |
|  | IFT172 |  | GOT2 |
|  | WDR19 |  | ARG1 |
|  | SNHG14 |  | CORIN |
|  | BRAF |  | HNF1B |
|  | MIR335 |  | CFI |
|  | IL1B |  | TRIM25 |
|  | MIR493HG |  | DGKE |
|  | ENSG00000276919 |  | CD46 |
|  | PLCE1 |  | CACNA1S |
|  | MPO |  | HELLPAR |
|  | LINC00520 |  | SLC26A1 |
|  | VEGFA |  | FLT1 |
|  | LINC-ROR |  | STOX1 |
|  | SLC4A1 |  | CCND1 |
|  | PAX2 |  | RYR1 |
|  | MIR155 |  | SAA1 |
|  | STAT3 |  | CFH |
|  | S100A9 |  | CFHR1 |
|  | CSF3 |  | HNF4A |
|  | CALCA |  | HPRT1 |
|  | C3AR1 |  | TCN2 |
|  | MIR451A |  | CFB |
|  | ABCB1 |  | CFHR3 |
|  | VWF |  | LPIN1 |
|  | SERPINE1 |  | OGA |
|  | IDH1 |  | SLC5A2 |
|  | CYLD |  | SCN7A |
|  | IKBKG |  | NBAS |
|  | MIR140 |  | MIR382 |
|  | MIR181A2 |  | NAGLU |
|  | MIR342 |  | FABP4 |
|  | MIRLET7E |  | CASP3 |
|  | MIR561 |  | SLC2A9 |
|  | CASP3 |  | BMP7 |
|  | CXCL9 |  | CPT2 |
|  | ATF3 |  | SLC2A6 |
|  | DANCR |  | ADAMTS13 |
|  | RAB4B-EGLN2 |  | MIOX |
|  | LMCD1-AS1 |  | PPARA |
|  | F3 |  | CCHCR1 |
|  | SERPINC1 |  | POR |
|  | YARS1 |  | ATM |
|  | IRF2 |  | MMP2 |
|  | TBX1 |  | CD40 |
|  | TIMP1 |  | PNPLA2 |
|  | AQP2 |  | SLC33A1 |
|  | FGFR3 |  | SELENBP1 |
|  | CDK4 |  | NR1I2 |
|  | CREBBP |  | CAT |
|  | PTPN11 |  | FGF23 |
|  | ATM |  | CAD |
|  | KRAS |  | SHBG |
|  | HDAC4 |  | IGAN1 |
|  | HRAS |  | ACHE |
|  | IDH2 |  | MBL2 |
|  | PIK3CA |  | SOSTDC1 |
|  | AURKA |  | STX2 |
|  | DNMT3A |  | AGTR1 |
|  | NRAS |  | DECR1 |
|  | PIK3R2 |  | CYBA |
|  | IKZF1 |  | PERCC1 |
|  | MAPKAPK3 |  | HYOU1 |
|  | RXRA |  | CDKN2A |
|  | SGK1 |  | IL17B |
|  | SLC2A2 |  | TOR2A |
|  | FCGR2B |  | GPX4 |
|  | BAP1 |  | MIR187 |
|  | BARD1 |  | KCNJ11 |
|  | FCGR2A |  | IL17A |
|  | LIG4 |  | CXCL8 |
|  | NF1 |  | IFNG |
|  | NKX2-1 |  | HGF |
|  | SH2B3 |  | PGR-AS1 |
|  | KDM5C |  |  |
|  | TCF3 |  |  |
|  | TET2 |  |  |
|  | CISH |  |  |
|  | IDUA |  |  |
|  | SMARCAL1 |  |  |
|  | CDKN2C |  |  |
|  | KMT2C |  |  |
|  | LATS1 |  |  |
|  | NSD1 |  |  |
|  | GYPA |  |  |
|  | GYPC |  |  |
|  | KMT2D |  |  |
|  | NCOR2 |  |  |
|  | SAMHD1 |  |  |
|  | SARDH |  |  |
|  | SLC12A4 |  |  |
|  | H1-4 |  |  |
|  | TIRAP |  |  |
|  | TRNT1 |  |  |
|  | ACKR1 |  |  |
|  | DIS3 |  |  |
|  | EIF1AX |  |  |
|  | ERF |  |  |
|  | RECQL4 |  |  |
|  | SHOC2 |  |  |
|  | TET3 |  |  |
|  | TRAF5 |  |  |
|  | POLG2 |  |  |
|  | DAAM2 |  |  |
|  | FAT1 |  |  |
|  | NCR3 |  |  |
|  | ARID4A |  |  |
|  | DYNC2H1 |  |  |
|  | H3C1 |  |  |
|  | RINT1 |  |  |
|  | MGA |  |  |
|  | GYPB |  |  |
|  | P2RY8 |  |  |
|  | LRRC56 |  |  |
|  | BCORL1 |  |  |
|  | FAM135A |  |  |
|  | H2AC16 |  |  |
|  | AXDND1 |  |  |
|  | CYS1 |  |  |
|  | H2AC17 |  |  |
|  | MILR1 |  |  |
|  | UCA1 |  |  |
|  | MIR32 |  |  |
|  | TP73-AS1 |  |  |
|  | MIR19A |  |  |
|  | MIR25 |  |  |
|  | MIR99B |  |  |
|  | PCAT1 |  |  |
|  | ST3GAL6-AS1 |  |  |
|  | TSIX |  |  |
|  | TET2-AS1 |  |  |
|  | LAMA5-AS1 |  |  |
|  | SPRY4-IT1 |  |  |
|  | PDIA3P1 |  |  |
|  | WFDC21P |  |  |
|  | LOC112694756 |  |  |
|  | YWHAEP7 |  |  |
|  | LOC105371046 |  |  |
|  | LIMASI |  |  |
|  | CEACAMP8 |  |  |
|  | FLT1P1 |  |  |
|  | LOC106099062 |  |  |
|  | LOC107032760 |  |  |
|  | LOC107133510 |  |  |
|  | LOC110006319 |  |  |
|  | LOC111365141 |  |  |
|  | LOC126806147 |  |  |
|  | LOC126862260 |  |  |
|  | LOC126862549 |  |  |
|  | LOC126859690 |  |  |
|  | DEL10Q26 |  |  |
|  | EPOR |  |  |
|  | SLK |  |  |
|  | FGA |  |  |
|  | MIR142 |  |  |
|  | TF |  |  |
|  | MIR133B |  |  |
|  | AGER |  |  |
|  | IFNG |  |  |
|  | MIR423 |  |  |
|  | TNFRSF1B |  |  |
|  | MBL2 |  |  |
|  | ANGPT1 |  |  |
|  | NFE2L2 |  |  |
|  | KCNQ1OT1 |  |  |
|  | BCL2 |  |  |
|  | HMGB1 |  |  |
|  | HLA-DRB1 |  |  |
|  | NEAT1 |  |  |
|  | CHI3L1 |  |  |
|  | AQP1 |  |  |
|  | LINC01618 |  |  |
|  | SMAD5-AS1 |  |  |
|  | MAP4K2 |  |  |
|  | INF2 |  |  |
|  | CYP3A5 |  |  |
|  | CASC2 |  |  |
|  | BSG |  |  |
|  | CCL3 |  |  |
|  | MIR210 |  |  |
|  | CTNNB1 |  |  |
|  | CDH1 |  |  |
|  | VDR |  |  |
|  | DLX6-AS1 |  |  |
|  | MIR199A1 |  |  |
|  | MIR146A |  |  |
|  | NPHS1 |  |  |
|  | MIRLET7C |  |  |
|  | MIR192 |  |  |
|  | SLC17A5 |  |  |
|  | SERPINA4 |  |  |
|  | SERPINA1 |  |  |
|  | VNN1 |  |  |
|  | IL5 |  |  |
|  | CXCL2 |  |  |
|  | PNMT |  |  |
|  | MIR376B |  |  |
|  | SLC12A1 |  |  |
|  | BMAL2 |  |  |
|  | LINC00963 |  |  |
|  | SLC6A6 |  |  |
|  | MMP9 |  |  |
|  | LOC110120845 |  |  |
|  | LINC00504 |  |  |
|  | TTTY10 |  |  |
|  | LINC02882 |  |  |
|  | ARF4-AS1 |  |  |
|  | LINC01258 |  |  |
|  | LINC01725 |  |  |
|  | LINC02955 |  |  |
|  | LINC02895 |  |  |
|  | ENSG00000230490 |  |  |
|  | ENSG00000245768 |  |  |
|  | ENSG00000250519 |  |  |
|  | ENSG00000258081 |  |  |
|  | ENSG00000235450 |  |  |
|  | ENSG00000251216 |  |  |
|  | LOC101927560 |  |  |
|  | ENSG00000253288 |  |  |
|  | lnc-IQCM-2 |  |  |
|  | lnc-HMGXB4-8 |  |  |
|  | MK280073-022 |  |  |
|  | MK280073-023 |  |  |
|  | MK280073-058 |  |  |
|  | MK280073-120 |  |  |
|  | MK280073-197 |  |  |
|  | MK280073-202 |  |  |
|  | MK280073-346 |  |  |
|  | MK280073-501 |  |  |
|  | MK280073-521 |  |  |
|  | MK280073-008 |  |  |
|  | MK280073-013 |  |  |
|  | MK280073-020 |  |  |
|  | MK280073-025 |  |  |
|  | MK280073-049 |  |  |
|  | MK280073-052 |  |  |
|  | MK280073-055 |  |  |
|  | MK280073-059 |  |  |
|  | MK280073-060 |  |  |
|  | MK280073-063 |  |  |
|  | MK280073-083 |  |  |
|  | MK280073-093 |  |  |
|  | MK280073-121 |  |  |
|  | MK280073-164 |  |  |
|  | MK280073-175 |  |  |
|  | MK280073-199 |  |  |
|  | MK280073-203 |  |  |
|  | MK280073-206 |  |  |
|  | MK280073-216 |  |  |
|  | MK280073-273 |  |  |
|  | MK280073-296 |  |  |
|  | MK280073-300 |  |  |
|  | MK280073-334 |  |  |
|  | MK280073-351 |  |  |
|  | MK280073-353 |  |  |
|  | MK280073-460 |  |  |
|  | MK280073-461 |  |  |
|  | MK280073-475 |  |  |
|  | MK280073-493 |  |  |
|  | MK280073-510 |  |  |
|  | MK280073-513 |  |  |
|  | MK280073-522 |  |  |
|  | MK280073-524 |  |  |
|  | MK280073-527 |  |  |
|  | MK280073-571 |  |  |
|  | MK280073-621 |  |  |
|  | MK280073-623 |  |  |
|  | MK280073-001 |  |  |
|  | MK280073-007 |  |  |
|  | MK280073-018 |  |  |
|  | MK280073-027 |  |  |
|  | MK280073-028 |  |  |
|  | MK280073-030 |  |  |
|  | MK280073-037 |  |  |
|  | MK280073-040 |  |  |
|  | MK280073-044 |  |  |
|  | MK280073-046 |  |  |
|  | MK280073-047 |  |  |
|  | MK280073-051 |  |  |
|  | MK280073-053 |  |  |
|  | MK280073-056 |  |  |
|  | MK280073-057 |  |  |
|  | MK280073-071 |  |  |
|  | MK280073-072 |  |  |
|  | MK280073-073 |  |  |
|  | MK280073-075 |  |  |
|  | MK280073-079 |  |  |
|  | MK280073-081 |  |  |
|  | MK280073-082 |  |  |
|  | MK280073-089 |  |  |
|  | MK280073-090 |  |  |
|  | MK280073-091 |  |  |
|  | MK280073-092 |  |  |
|  | MK280073-103 |  |  |
|  | MK280073-106 |  |  |
|  | MK280073-115 |  |  |
|  | MK280073-116 |  |  |
|  | MK280073-117 |  |  |
|  | MK280073-119 |  |  |
|  | MK280073-123 |  |  |
|  | MK280073-125 |  |  |
|  | MK280073-140 |  |  |
|  | MK280073-149 |  |  |
|  | MK280073-150 |  |  |
|  | MK280073-153 |  |  |
|  | MK280073-154 |  |  |
|  | MK280073-168 |  |  |
|  | MK280073-174 |  |  |
|  | MK280073-176 |  |  |
|  | MK280073-179 |  |  |
|  | MK280073-180 |  |  |
|  | MK280073-182 |  |  |
|  | MK280073-183 |  |  |
|  | MK280073-185 |  |  |
|  | MK280073-190 |  |  |
|  | MK280073-194 |  |  |
|  | MK280073-205 |  |  |
|  | MK280073-207 |  |  |
|  | MK280073-208 |  |  |
|  | MK280073-221 |  |  |
|  | MK280073-233 |  |  |
|  | MK280073-243 |  |  |
|  | MK280073-247 |  |  |
|  | MK280073-258 |  |  |
|  | MK280073-280 |  |  |
|  | MK280073-283 |  |  |
|  | MK280073-295 |  |  |
|  | MK280073-301 |  |  |
|  | MK280073-302 |  |  |
|  | MK280073-313 |  |  |
|  | MK280073-345 |  |  |
|  | MK280073-349 |  |  |
|  | MK280073-352 |  |  |
|  | MK280073-354 |  |  |
|  | MK280073-355 |  |  |
|  | MK280073-357 |  |  |
|  | MK280073-359 |  |  |
|  | MK280073-364 |  |  |
|  | MK280073-384 |  |  |
|  | MK280073-389 |  |  |
|  | MK280073-396 |  |  |
|  | MK280073-397 |  |  |
|  | MK280073-414 |  |  |
|  | MK280073-434 |  |  |
|  | MK280073-445 |  |  |
|  | MK280073-453 |  |  |
|  | MK280073-455 |  |  |
|  | MK280073-456 |  |  |
|  | MK280073-464 |  |  |
|  | MK280073-465 |  |  |
|  | MK280073-466 |  |  |
|  | MK280073-467 |  |  |
|  | MK280073-468 |  |  |
|  | MK280073-470 |  |  |
|  | MK280073-473 |  |  |
|  | MK280073-476 |  |  |
|  | MK280073-486 |  |  |
|  | MK280073-487 |  |  |
|  | MK280073-490 |  |  |
|  | MK280073-495 |  |  |
|  | MK280073-496 |  |  |
|  | MK280073-499 |  |  |
|  | MK280073-500 |  |  |
|  | MK280073-512 |  |  |
|  | MK280073-515 |  |  |
|  | MK280073-516 |  |  |
|  | MK280073-519 |  |  |
|  | MK280073-523 |  |  |
|  | MK280073-525 |  |  |
|  | MK280073-530 |  |  |
|  | MK280073-531 |  |  |
|  | MK280073-532 |  |  |
|  | MK280073-533 |  |  |
|  | MK280073-538 |  |  |
|  | MK280073-540 |  |  |
|  | MK280073-543 |  |  |
|  | MK280073-544 |  |  |
|  | MK280073-551 |  |  |
|  | MK280073-554 |  |  |
|  | MK280073-556 |  |  |
|  | MK280073-557 |  |  |
|  | MK280073-558 |  |  |
|  | MK280073-559 |  |  |
|  | MK280073-561 |  |  |
|  | MK280073-564 |  |  |
|  | MK280073-574 |  |  |
|  | MK280073-578 |  |  |
|  | MK280073-585 |  |  |
|  | MK280073-586 |  |  |
|  | MK280073-590 |  |  |
|  | MK280073-592 |  |  |
|  | MK280073-593 |  |  |
|  | MK280073-596 |  |  |
|  | MK280073-597 |  |  |
|  | MK280073-609 |  |  |
|  | MK280073-618 |  |  |
|  | MK280073-655 |  |  |
|  | MK280073-659 |  |  |
|  | MK280073-688 |  |  |
|  | MK280073-689 |  |  |
|  | lnc-LRP5L-15 |  |  |
|  | MK280073-002 |  |  |
|  | MK280073-003 |  |  |
|  | MK280073-004 |  |  |
|  | MK280073-005 |  |  |
|  | MK280073-006 |  |  |
|  | MK280073-009 |  |  |
|  | MK280073-010 |  |  |
|  | MK280073-011 |  |  |
|  | MK280073-012 |  |  |
|  | MK280073-014 |  |  |
|  | MK280073-015 |  |  |
|  | MK280073-016 |  |  |
|  | MK280073-017 |  |  |
|  | MK280073-019 |  |  |
|  | MK280073-021 |  |  |
|  | MK280073-024 |  |  |
|  | MK280073-026 |  |  |
|  | MK280073-029 |  |  |
|  | MK280073-031 |  |  |
|  | MK280073-032 |  |  |
|  | MK280073-033 |  |  |
|  | MK280073-034 |  |  |
|  | MK280073-035 |  |  |
|  | MK280073-036 |  |  |
|  | MK280073-038 |  |  |
|  | MK280073-039 |  |  |
|  | MK280073-041 |  |  |
|  | MK280073-042 |  |  |
|  | MK280073-043 |  |  |
|  | MK280073-045 |  |  |
|  | MK280073-048 |  |  |
|  | MK280073-050 |  |  |
|  | MK280073-054 |  |  |
|  | MK280073-061 |  |  |
|  | MK280073-062 |  |  |
|  | MK280073-064 |  |  |
|  | MK280073-065 |  |  |
|  | MK280073-066 |  |  |
|  | MK280073-068 |  |  |
|  | MK280073-069 |  |  |
|  | MK280073-070 |  |  |
|  | MK280073-074 |  |  |
|  | MK280073-076 |  |  |
|  | MK280073-077 |  |  |
|  | MK280073-078 |  |  |
|  | MK280073-080 |  |  |
|  | MK280073-084 |  |  |
|  | MK280073-085 |  |  |
|  | MK280073-086 |  |  |
|  | MK280073-087 |  |  |
|  | MK280073-088 |  |  |
|  | MK280073-094 |  |  |
|  | MK280073-095 |  |  |
|  | MK280073-096 |  |  |
|  | MK280073-097 |  |  |
|  | MK280073-098 |  |  |
|  | MK280073-101 |  |  |
|  | MK280073-102 |  |  |
|  | MK280073-104 |  |  |
|  | MK280073-105 |  |  |
|  | MK280073-107 |  |  |
|  | MK280073-108 |  |  |
|  | MK280073-109 |  |  |
|  | MK280073-110 |  |  |
|  | MK280073-111 |  |  |
|  | MK280073-112 |  |  |
|  | MK280073-113 |  |  |
|  | MK280073-114 |  |  |
|  | MK280073-118 |  |  |
|  | MK280073-122 |  |  |
|  | MK280073-124 |  |  |
|  | MK280073-126 |  |  |
|  | MK280073-127 |  |  |
|  | MK280073-128 |  |  |
|  | MK280073-131 |  |  |
|  | MK280073-132 |  |  |
|  | MK280073-133 |  |  |
|  | MK280073-134 |  |  |
|  | MK280073-135 |  |  |
|  | MK280073-136 |  |  |
|  | MK280073-137 |  |  |
|  | MK280073-138 |  |  |
|  | MK280073-139 |  |  |
|  | MK280073-141 |  |  |
|  | MK280073-142 |  |  |
|  | MK280073-143 |  |  |
|  | MK280073-144 |  |  |
|  | MK280073-145 |  |  |
|  | MK280073-146 |  |  |
|  | MK280073-147 |  |  |
|  | MK280073-148 |  |  |
|  | MK280073-151 |  |  |
|  | MK280073-152 |  |  |
|  | MK280073-155 |  |  |
|  | MK280073-156 |  |  |
|  | MK280073-157 |  |  |
|  | MK280073-158 |  |  |
|  | MK280073-159 |  |  |
|  | MK280073-160 |  |  |
|  | MK280073-161 |  |  |
|  | MK280073-162 |  |  |
|  | MK280073-163 |  |  |
|  | MK280073-165 |  |  |
|  | MK280073-169 |  |  |
|  | MK280073-170 |  |  |
|  | MK280073-171 |  |  |
|  | MK280073-172 |  |  |
|  | MK280073-173 |  |  |
|  | MK280073-177 |  |  |
|  | MK280073-178 |  |  |
|  | MK280073-181 |  |  |
|  | MK280073-184 |  |  |
|  | MK280073-186 |  |  |
|  | MK280073-187 |  |  |
|  | MK280073-188 |  |  |
|  | MK280073-189 |  |  |
|  | MK280073-191 |  |  |
|  | MK280073-192 |  |  |
|  | MK280073-193 |  |  |
|  | MK280073-195 |  |  |
|  | MK280073-196 |  |  |
|  | MK280073-198 |  |  |
|  | MK280073-200 |  |  |
|  | MK280073-201 |  |  |
|  | MK280073-204 |  |  |
|  | MK280073-209 |  |  |
|  | MK280073-210 |  |  |
|  | MK280073-211 |  |  |
|  | MK280073-212 |  |  |
|  | MK280073-213 |  |  |
|  | MK280073-214 |  |  |
|  | MK280073-215 |  |  |
|  | MK280073-217 |  |  |
|  | MK280073-218 |  |  |
|  | MK280073-219 |  |  |
|  | MK280073-220 |  |  |
|  | MK280073-222 |  |  |
|  | MK280073-224 |  |  |
|  | MK280073-225 |  |  |
|  | MK280073-226 |  |  |
|  | MK280073-227 |  |  |
|  | MK280073-228 |  |  |
|  | MK280073-229 |  |  |
|  | MK280073-230 |  |  |
|  | MK280073-231 |  |  |
|  | MK280073-232 |  |  |
|  | MK280073-234 |  |  |
|  | MK280073-235 |  |  |
|  | MK280073-236 |  |  |
|  | MK280073-237 |  |  |
|  | MK280073-238 |  |  |
|  | MK280073-239 |  |  |
|  | MK280073-240 |  |  |
|  | MK280073-241 |  |  |
|  | MK280073-242 |  |  |
|  | MK280073-244 |  |  |
|  | MK280073-245 |  |  |
|  | MK280073-246 |  |  |
|  | MK280073-248 |  |  |
|  | MK280073-249 |  |  |
|  | MK280073-250 |  |  |
|  | MK280073-251 |  |  |
|  | MK280073-252 |  |  |
|  | MK280073-253 |  |  |
|  | MK280073-255 |  |  |
|  | MK280073-257 |  |  |
|  | MK280073-259 |  |  |
|  | MK280073-260 |  |  |
|  | MK280073-261 |  |  |
|  | MK280073-262 |  |  |
|  | MK280073-264 |  |  |
|  | MK280073-265 |  |  |
|  | MK280073-266 |  |  |
|  | MK280073-267 |  |  |
|  | MK280073-268 |  |  |
|  | MK280073-269 |  |  |
|  | MK280073-270 |  |  |
|  | MK280073-271 |  |  |
|  | MK280073-272 |  |  |
|  | MK280073-274 |  |  |
|  | MK280073-275 |  |  |
|  | MK280073-276 |  |  |
|  | MK280073-277 |  |  |
|  | MK280073-278 |  |  |
|  | MK280073-279 |  |  |
|  | MK280073-281 |  |  |
|  | MK280073-282 |  |  |
|  | MK280073-284 |  |  |
|  | MK280073-285 |  |  |
|  | MK280073-286 |  |  |
|  | MK280073-287 |  |  |
|  | MK280073-288 |  |  |
|  | MK280073-289 |  |  |
|  | MK280073-290 |  |  |
|  | MK280073-291 |  |  |
|  | MK280073-292 |  |  |
|  | MK280073-293 |  |  |
|  | MK280073-294 |  |  |
|  | MK280073-297 |  |  |
|  | MK280073-298 |  |  |
|  | MK280073-299 |  |  |
|  | MK280073-303 |  |  |
|  | MK280073-304 |  |  |
|  | MK280073-305 |  |  |
|  | MK280073-306 |  |  |
|  | MK280073-307 |  |  |
|  | MK280073-308 |  |  |
|  | MK280073-309 |  |  |
|  | MK280073-310 |  |  |
|  | MK280073-311 |  |  |
|  | MK280073-312 |  |  |
|  | MK280073-314 |  |  |
|  | MK280073-315 |  |  |
|  | MK280073-316 |  |  |
|  | MK280073-317 |  |  |
|  | MK280073-318 |  |  |
|  | MK280073-319 |  |  |
|  | MK280073-320 |  |  |
|  | MK280073-321 |  |  |
|  | MK280073-322 |  |  |
|  | MK280073-323 |  |  |
|  | MK280073-324 |  |  |
|  | MK280073-325 |  |  |
|  | MK280073-326 |  |  |
|  | MK280073-328 |  |  |
|  | MK280073-329 |  |  |
|  | MK280073-330 |  |  |
|  | MK280073-331 |  |  |
|  | MK280073-332 |  |  |
|  | MK280073-333 |  |  |
|  | MK280073-335 |  |  |
|  | MK280073-336 |  |  |
|  | MK280073-337 |  |  |
|  | MK280073-338 |  |  |
|  | MK280073-339 |  |  |
|  | MK280073-340 |  |  |
|  | MK280073-341 |  |  |
|  | MK280073-342 |  |  |
|  | MK280073-343 |  |  |
|  | MK280073-344 |  |  |
|  | MK280073-347 |  |  |
|  | MK280073-348 |  |  |
|  | MK280073-350 |  |  |
|  | MK280073-356 |  |  |
|  | MK280073-358 |  |  |
|  | MK280073-360 |  |  |
|  | MK280073-361 |  |  |
|  | MK280073-362 |  |  |
|  | MK280073-363 |  |  |
|  | MK280073-365 |  |  |
|  | MK280073-366 |  |  |
|  | MK280073-367 |  |  |
|  | MK280073-368 |  |  |
|  | MK280073-369 |  |  |
|  | MK280073-370 |  |  |
|  | MK280073-371 |  |  |
|  | MK280073-372 |  |  |
|  | MK280073-373 |  |  |
|  | MK280073-374 |  |  |
|  | MK280073-375 |  |  |
|  | MK280073-376 |  |  |
|  | MK280073-377 |  |  |
|  | MK280073-378 |  |  |
|  | MK280073-379 |  |  |
|  | MK280073-380 |  |  |
|  | MK280073-381 |  |  |
|  | MK280073-382 |  |  |
|  | MK280073-383 |  |  |
|  | MK280073-385 |  |  |
|  | MK280073-386 |  |  |
|  | MK280073-387 |  |  |
|  | MK280073-388 |  |  |
|  | MK280073-390 |  |  |
|  | MK280073-391 |  |  |
|  | MK280073-395 |  |  |
|  | MK280073-398 |  |  |
|  | MK280073-399 |  |  |
|  | MK280073-402 |  |  |
|  | MK280073-403 |  |  |
|  | MK280073-404 |  |  |
|  | MK280073-405 |  |  |
|  | MK280073-406 |  |  |
|  | MK280073-407 |  |  |
|  | MK280073-409 |  |  |
|  | MK280073-410 |  |  |
|  | MK280073-411 |  |  |
|  | MK280073-412 |  |  |
|  | MK280073-413 |  |  |
|  | MK280073-415 |  |  |
|  | MK280073-416 |  |  |
|  | MK280073-417 |  |  |
|  | MK280073-418 |  |  |
|  | MK280073-419 |  |  |
|  | MK280073-420 |  |  |
|  | MK280073-427 |  |  |
|  | MK280073-428 |  |  |
|  | MK280073-430 |  |  |
|  | MK280073-431 |  |  |
|  | MK280073-432 |  |  |
|  | MK280073-433 |  |  |
|  | MK280073-435 |  |  |
|  | MK280073-436 |  |  |
|  | MK280073-437 |  |  |
|  | MK280073-438 |  |  |
|  | MK280073-439 |  |  |
|  | MK280073-440 |  |  |
|  | MK280073-441 |  |  |
|  | MK280073-442 |  |  |
|  | MK280073-443 |  |  |
|  | MK280073-444 |  |  |
|  | MK280073-446 |  |  |
|  | MK280073-447 |  |  |
|  | MK280073-448 |  |  |
|  | MK280073-449 |  |  |
|  | MK280073-450 |  |  |
|  | MK280073-451 |  |  |
|  | MK280073-452 |  |  |
|  | MK280073-454 |  |  |
|  | MK280073-457 |  |  |
|  | MK280073-458 |  |  |
|  | MK280073-459 |  |  |
|  | MK280073-462 |  |  |
|  | MK280073-463 |  |  |
|  | MK280073-469 |  |  |
|  | MK280073-471 |  |  |
|  | MK280073-472 |  |  |
|  | MK280073-474 |  |  |
|  | MK280073-477 |  |  |
|  | MK280073-478 |  |  |
|  | MK280073-479 |  |  |
|  | MK280073-480 |  |  |
|  | MK280073-481 |  |  |
|  | MK280073-482 |  |  |
|  | MK280073-483 |  |  |
|  | MK280073-484 |  |  |
|  | MK280073-485 |  |  |
|  | MK280073-488 |  |  |
|  | MK280073-491 |  |  |
|  | MK280073-492 |  |  |
|  | MK280073-494 |  |  |
|  | MK280073-497 |  |  |
|  | MK280073-498 |  |  |
|  | MK280073-502 |  |  |
|  | MK280073-503 |  |  |
|  | MK280073-504 |  |  |
|  | MK280073-505 |  |  |
|  | MK280073-506 |  |  |
|  | MK280073-507 |  |  |
|  | MK280073-508 |  |  |
|  | MK280073-509 |  |  |
|  | MK280073-511 |  |  |
|  | MK280073-514 |  |  |
|  | MK280073-517 |  |  |
|  | MK280073-518 |  |  |
|  | MK280073-526 |  |  |
|  | MK280073-528 |  |  |
|  | MK280073-529 |  |  |
|  | MK280073-534 |  |  |
|  | MK280073-535 |  |  |
|  | MK280073-536 |  |  |
|  | MK280073-537 |  |  |
|  | MK280073-541 |  |  |
|  | MK280073-542 |  |  |
|  | MK280073-545 |  |  |
|  | MK280073-546 |  |  |
|  | MK280073-547 |  |  |
|  | MK280073-548 |  |  |
|  | MK280073-549 |  |  |
|  | MK280073-550 |  |  |
|  | MK280073-552 |  |  |
|  | MK280073-553 |  |  |
|  | MK280073-555 |  |  |
|  | MK280073-560 |  |  |
|  | MK280073-562 |  |  |
|  | MK280073-563 |  |  |
|  | MK280073-565 |  |  |
|  | MK280073-566 |  |  |
|  | MK280073-567 |  |  |
|  | MK280073-568 |  |  |
|  | MK280073-569 |  |  |
|  | MK280073-570 |  |  |
|  | MK280073-572 |  |  |
|  | MK280073-573 |  |  |
|  | MK280073-575 |  |  |
|  | MK280073-577 |  |  |
|  | MK280073-579 |  |  |
|  | MK280073-580 |  |  |
|  | MK280073-581 |  |  |
|  | MK280073-582 |  |  |
|  | MK280073-583 |  |  |
|  | MK280073-584 |  |  |
|  | MK280073-588 |  |  |
|  | MK280073-589 |  |  |
|  | MK280073-591 |  |  |
|  | MK280073-594 |  |  |
|  | MK280073-595 |  |  |
|  | MK280073-598 |  |  |
|  | MK280073-606 |  |  |
|  | MK280073-615 |  |  |
|  | MK280073-624 |  |  |
|  | MK280073-625 |  |  |
|  | MK280073-629 |  |  |
|  | MK280073-654 |  |  |
|  | MK280073-656 |  |  |
|  | MK280073-670 |  |  |
|  | MK280073-674 |  |  |
|  | MK280073-676 |  |  |
|  | MK280073-678 |  |  |
|  | MK280073-683 |  |  |
|  | MK280073-686 |  |  |
|  | MK280073-691 |  |  |
|  | MK280073-067 |  |  |
|  | MK280073-099 |  |  |
|  | MK280073-100 |  |  |
|  | MK280073-129 |  |  |
|  | MK280073-130 |  |  |
|  | MK280073-166 |  |  |
|  | MK280073-167 |  |  |
|  | MK280073-223 |  |  |
|  | MK280073-254 |  |  |
|  | MK280073-256 |  |  |
|  | MK280073-263 |  |  |
|  | MK280073-327 |  |  |
|  | MK280073-392 |  |  |
|  | MK280073-393 |  |  |
|  | MK280073-394 |  |  |
|  | MK280073-400 |  |  |
|  | MK280073-401 |  |  |
|  | MK280073-408 |  |  |
|  | MK280073-421 |  |  |
|  | MK280073-422 |  |  |
|  | MK280073-423 |  |  |
|  | MK280073-424 |  |  |
|  | MK280073-425 |  |  |
|  | MK280073-426 |  |  |
|  | MK280073-429 |  |  |
|  | MK280073-489 |  |  |
|  | MK280073-520 |  |  |
|  | MK280073-539 |  |  |
|  | MK280073-576 |  |  |
|  | MK280073-587 |  |  |
|  | MK280073-599 |  |  |
|  | MK280073-600 |  |  |
|  | MK280073-601 |  |  |
|  | MK280073-602 |  |  |
|  | MK280073-603 |  |  |
|  | MK280073-604 |  |  |
|  | MK280073-605 |  |  |
|  | MK280073-607 |  |  |
|  | MK280073-608 |  |  |
|  | MK280073-610 |  |  |
|  | MK280073-611 |  |  |
|  | MK280073-612 |  |  |
|  | MK280073-613 |  |  |
|  | MK280073-614 |  |  |
|  | MK280073-616 |  |  |
|  | MK280073-617 |  |  |
|  | MK280073-619 |  |  |
|  | MK280073-620 |  |  |
|  | MK280073-622 |  |  |
|  | MK280073-626 |  |  |
|  | MK280073-627 |  |  |
|  | MK280073-628 |  |  |
|  | MK280073-630 |  |  |
|  | MK280073-631 |  |  |
|  | MK280073-632 |  |  |
|  | MK280073-633 |  |  |
|  | MK280073-634 |  |  |
|  | MK280073-635 |  |  |
|  | MK280073-636 |  |  |
|  | MK280073-637 |  |  |
|  | MK280073-638 |  |  |
|  | MK280073-639 |  |  |
|  | MK280073-640 |  |  |
|  | MK280073-641 |  |  |
|  | MK280073-642 |  |  |
|  | MK280073-643 |  |  |
|  | MK280073-644 |  |  |
|  | MK280073-645 |  |  |
|  | MK280073-646 |  |  |
|  | MK280073-647 |  |  |
|  | MK280073-648 |  |  |
|  | MK280073-649 |  |  |
|  | MK280073-650 |  |  |
|  | MK280073-651 |  |  |
|  | MK280073-652 |  |  |
|  | MK280073-653 |  |  |
|  | MK280073-657 |  |  |
|  | MK280073-658 |  |  |
|  | MK280073-660 |  |  |
|  | MK280073-661 |  |  |
|  | MK280073-662 |  |  |
|  | MK280073-663 |  |  |
|  | MK280073-664 |  |  |
|  | MK280073-665 |  |  |
|  | MK280073-666 |  |  |
|  | MK280073-667 |  |  |
|  | MK280073-668 |  |  |
|  | MK280073-669 |  |  |
|  | MK280073-671 |  |  |
|  | MK280073-672 |  |  |
|  | MK280073-673 |  |  |
|  | MK280073-675 |  |  |
|  | MK280073-677 |  |  |
|  | MK280073-679 |  |  |
|  | MK280073-680 |  |  |
|  | MK280073-681 |  |  |
|  | MK280073-682 |  |  |
|  | MK280073-684 |  |  |
|  | MK280073-685 |  |  |
|  | MK280073-687 |  |  |
|  | MK280073-690 |  |  |
|  | MK280073-692 |  |  |
|  | MK280073-693 |  |  |
|  | MK280073-694 |  |  |
|  | MK280073-695 |  |  |
|  | MK280073-696 |  |  |
|  | MK280073-697 |  |  |
|  | MK280073-698 |  |  |
|  | MK280073-699 |  |  |
|  | MK280073-700 |  |  |
|  | MK280073-701 |  |  |
|  | MK280073-702 |  |  |
|  | MK280073-703 |  |  |
|  | MK280073-704 |  |  |
|  | MK280073-705 |  |  |
|  | MK280073-706 |  |  |
|  | MK280073-707 |  |  |
|  | MK280073-708 |  |  |
|  | MK280073-709 |  |  |
|  | MK280073-710 |  |  |
|  | MK280073-711 |  |  |
|  | MK280073-712 |  |  |
|  | MK280073-713 |  |  |
|  | MK280073-714 |  |  |
|  | MK280073-715 |  |  |
|  | MK280073-716 |  |  |
|  | MK280073-717 |  |  |
|  | MK280073-718 |  |  |
|  | MK280073-719 |  |  |
|  | MK280073-720 |  |  |
|  | MK280073-721 |  |  |
|  | MK280073-722 |  |  |
|  | IL4 |  |  |
|  | LRP2 |  |  |
|  | NFKB1 |  |  |
|  | SNORD118 |  |  |
|  | PLAUR |  |  |
|  | TNFSF12 |  |  |
|  | SNHG5 |  |  |
|  | IL2RA |  |  |
|  | IGFBP1 |  |  |
|  | MIR150 |  |  |
|  | SFTPD |  |  |
|  | IL2 |  |  |
|  | IL33 |  |  |
|  | CASP1 |  |  |
|  | TMPRSS2 |  |  |
|  | CCL4 |  |  |
|  | TREM1 |  |  |
|  | MIR216A |  |  |
|  | INS |  |  |
|  | SNORD15A |  |  |
|  | TRAF6 |  |  |
|  | CXCR3 |  |  |
|  | CXCL11 |  |  |
|  | C5 |  |  |
|  | HSPB1 |  |  |
|  | FABP3 |  |  |
|  | XDH |  |  |
|  | MIAT |  |  |
|  | ADCYAP1 |  |  |
|  | HPSE |  |  |
|  | AGO2 |  |  |
|  | MIR30A |  |  |
|  | SQSTM1 |  |  |
|  | TLX1NB |  |  |
|  | MIR495 |  |  |
|  | TRE-TTC3-1 |  |  |
|  | RIPK3 |  |  |
|  | SLC22A2 |  |  |
|  | SLC22A8 |  |  |
|  | GSDME |  |  |
|  | LINC01191 |  |  |
|  | CYP3A4 |  |  |
|  | AGXT |  |  |
|  | FCGR1A |  |  |
|  | GSTP1 |  |  |
|  | NFKBIA |  |  |
|  | TNFRSF11B |  |  |
|  | ADIPOQ |  |  |
|  | FGF2 |  |  |
|  | CD163 |  |  |
|  | ADCY10 |  |  |
|  | KDM4C |  |  |
|  | COLEC11 |  |  |
|  | GRHPR |  |  |
|  | KLK1 |  |  |
|  | PPARA |  |  |
|  | HJV |  |  |
|  | KLF6 |  |  |
|  | MIR22 |  |  |
|  | MIR23A |  |  |
|  | MIR211 |  |  |
|  | ATG7 |  |  |
|  | MMP2 |  |  |
|  | HSPA5 |  |  |
|  | ADORA2A |  |  |
|  | CD14 |  |  |
|  | IRF1 |  |  |
|  | LNPEP |  |  |
|  | PAPPA |  |  |
|  | AIM2 |  |  |
|  | BBC3 |  |  |
|  | MIR126 |  |  |
|  | MIR17 |  |  |
|  | MIR215 |  |  |
|  | SOD1 |  |  |
|  | PARP1 |  |  |
|  | HDAC6 |  |  |
|  | TNC |  |  |
|  | C1orf54 |  |  |
|  | MIR204 |  |  |
|  | MIR141 |  |  |
|  | MIR191 |  |  |
|  | MIR34B |  |  |
|  | SELP |  |  |
|  | MASP2 |  |  |
|  | PTEN |  |  |
|  | LINC01629 |  |  |
|  | PTGS2 |  |  |
|  | CYP2B6 |  |  |
|  | HOXA11-AS |  |  |
|  | ENSG00000277966 |  |  |
|  | ENSG00000278592 |  |  |
|  | ENSG00000273961 |  |  |
|  | ENSG00000276496 |  |  |
|  | ENSG00000278020 |  |  |
|  | ENSG00000278334 |  |  |
|  | FOXM1 |  |  |
|  | MIR10A |  |  |
|  | IFNA1 |  |  |
|  | CAT |  |  |
|  | SEMA3A |  |  |
|  | MIR205 |  |  |
|  | SNHG16 |  |  |
|  | GGT1 |  |  |
|  | PPARGC1A |  |  |
|  | ZFAS1 |  |  |
|  | SLC2A9-AS1 |  |  |
|  | SOX9 |  |  |
|  | CDK5RAP3 |  |  |
|  | SCARB1 |  |  |
|  | STAT5A |  |  |
|  | SCARB2 |  |  |
|  | BMP6 |  |  |
|  | CASC9 |  |  |
|  | DARS1-AS1 |  |  |
|  | LOC101927394 |  |  |
|  | GSTM1 |  |  |
|  | ABCG2 |  |  |
|  | CASP4 |  |  |
|  | SIRT6 |  |  |
|  | NOX4 |  |  |
|  | TRAPPC10 |  |  |
|  | HMGCR |  |  |
|  | HSPA8 |  |  |
|  | LRG1 |  |  |
|  | SMAD3 |  |  |
|  | MAPK14 |  |  |
|  | AGRN |  |  |
|  | ATG16L1 |  |  |
|  | MIR30C1 |  |  |
|  | MIR30C2 |  |  |
|  | GC |  |  |
|  | FOXO1 |  |  |
|  | CHEK1 |  |  |
|  | SHH |  |  |
|  | APOH |  |  |
|  | BAX |  |  |
|  | MTHFR |  |  |
|  | WNT4 |  |  |
|  | DKK3 |  |  |
|  | ZNF644 |  |  |
|  | SPP1 |  |  |
|  | CD274 |  |  |
|  | ENSG00000274760 |  |  |
|  | ENSG00000277967 |  |  |
|  | TRIM21 |  |  |
|  | RELA |  |  |
|  | CASP5 |  |  |
|  | MIR18A |  |  |
|  | PTGS1 |  |  |
|  | AGTR2 |  |  |
|  | ABCC2 |  |  |
|  | SOD2 |  |  |
|  | MIR223 |  |  |
|  | EMSLR |  |  |
|  | CDKN1A |  |  |
|  | CCN2 |  |  |
|  | PROC |  |  |
|  | MIR545 |  |  |
|  | MMP8 |  |  |
|  | FN1 |  |  |
|  | CD79A |  |  |
|  | SELE |  |  |
|  | PLAT |  |  |
|  | IFNA2 |  |  |
|  | CCR6 |  |  |
|  | SLIT2 |  |  |
|  | ROBO1 |  |  |
|  | SPHK2 |  |  |
|  | RMRP |  |  |
|  | MIR20A |  |  |
|  | ST2 |  |  |
|  | MEG8 |  |  |
|  | IL22 |  |  |
|  | SDC1 |  |  |
|  | FTH1 |  |  |
|  | TNNI3 |  |  |
|  | RETN |  |  |
|  | S100A8 |  |  |
|  | COMMD5 |  |  |
|  | MIR381 |  |  |
|  | MIR4270 |  |  |
|  | PANX1 |  |  |
|  | STING1 |  |  |
|  | CD5L |  |  |
|  | HSPB2 |  |  |
|  | NLRP6 |  |  |
|  | CGAS |  |  |
|  | HEXD |  |  |
|  | MIR200C |  |  |
|  | MIR214 |  |  |
|  | MIR429 |  |  |
|  | OIP5-AS1 |  |  |
|  | MIR526B |  |  |
|  | TRL-TAG1-1 |  |  |
|  | MIR486-1 |  |  |
|  | RAPGEF1 |  |  |
|  | CLU |  |  |
|  | COL4A3 |  |  |
|  | EZH2 |  |  |
|  | PPARG |  |  |
|  | SLC2A1 |  |  |
|  | GSK3B |  |  |
|  | EPHX2 |  |  |
|  | NR1H4 |  |  |
|  | AQP5 |  |  |
|  | AVP |  |  |
|  | MMP10 |  |  |
|  | SERPINA5 |  |  |
|  | CDKL5 |  |  |
|  | CX3CL1 |  |  |
|  | MDK |  |  |
|  | GABARAP |  |  |
|  | PICK1 |  |  |
|  | PYCARD |  |  |
|  | RCAN1 |  |  |
|  | BCL3 |  |  |
|  | HPX |  |  |
|  | TNFSF14 |  |  |
|  | TNK1 |  |  |
|  | DYNLT1 |  |  |
|  | INTU |  |  |
|  | TIFA |  |  |
|  | LINC00261 |  |  |
|  | MIR122 |  |  |
|  | MIR27B |  |  |
|  | MIR370 |  |  |
|  | MIR574 |  |  |
|  | MIR199B |  |  |
|  | NORAD |  |  |
|  | MIR129-1 |  |  |
|  | RNY3 |  |  |
|  | MIR1184-1 |  |  |
|  | HDAC9 |  |  |
|  | CCL5 |  |  |
|  | PRKAG2 |  |  |
|  | DAXX |  |  |
|  | ITGAM |  |  |
|  | IL3 |  |  |
|  | F5 |  |  |
|  | ADM |  |  |
|  | CXCL12 |  |  |
|  | MIR27A |  |  |
|  | PWAR1 |  |  |
|  | MIR152 |  |  |
