# Supplementary material for: Tripterygium wilfordii Hook.f induced kidney injury through mediating inflammation via PI3K-Akt/HIF-1/TNF signaling pathway: A study of network toxicology and molecular docking
Source: Medicine (Baltimore). 2024 Feb 9;103(6):e36968. doi: 10.1097/MD.0000000000036968 (PMC10860970; doi:10.1097/MD.0000000000036968)
Supplement: Supplementary file 4 [file medi-103-e36968-s004.docx]

Supplemental Table 4 62 TwHF induced KI targets.

SHBG

HMGCR

AR

CYP2C19

ACHE

G6PD

NOS2

PPARG

NOX4

FLT3

ALOX5

ABCB1

ABCG2

GSK3B

MMP9

MMP2

ADORA2A

ARG1

SLC22A12

TTR

EGFR

IGF1R

F2

MPO

MET

PARP1

PTGS2

PPARA

PTGS1

SLC5A2

RXRA

NR1H4

MMP8

BRAF

AURKA

SGK1

TNF

CCND1

AGTR1

SIRT1

CDK4

GSTM1

CASP1

CAPN1

NFKBIA

RELA

MAPK14

KDM5C

HDAC6

EDNRB

CHEK1

ADCY10

PIK3CA

MTOR

ACE

HLA-DRB1

IDH1

FLT1

HIF1A

SHH

XDH

VDR
