# Supplementary material for: Tripterygium wilfordii Hook.f induced kidney injury through mediating inflammation via PI3K-Akt/HIF-1/TNF signaling pathway: A study of network toxicology and molecular docking
Source: Medicine (Baltimore). 2024 Feb 9;103(6):e36968. doi: 10.1097/MD.0000000000036968 (PMC10860970; doi:10.1097/MD.0000000000036968)
Supplement: Supplementary file 5 [file medi-103-e36968-s005.docx]

Supplemental Table 5 The specific expression results of 62 genes.

|  | **Specific expression (organ/tissue/cell)** | | |
| --- | --- | --- | --- |
| SHBG | NA |  |  |
| HMGCR | bronchial epithelial cells | 721 B lymphoblasts | colon |
| AR | liver | prostate |  |
| CYP2C19 | liver |  |  |
| ACHE | pineal night | pineal day | adipocyte |
| G6PD | whole blood | CD56+ NK cells | CD33+ myeloid |
| NOS2 | NA |  |  |
| PPARG | adipocyte |  |  |
| NOX4 | NA |  |  |
| FLT3 | BDCA4+ dentritic cells | CD34+ |  |
| ALOX5 | NA |  |  |
| ABCB1 | adrenal cortex | adrenal gland | CD19+ B cells |
| ABCG2 | CD71+ early erythroid | placenta | small intestine |
| GSK3B | bronchial epithelial cells | testis interstitial |  |
| MMP9 | bonemarrow | lymph node | tonsil |
| MMP2 | smooth muscle | adipocyte | uterus corpus |
| ADORA2A | thymus | caudate nucleus |  |
| ARG1 | fetal liver | CD71+ early erythroid | liver |
| SLC22A12 | NA |  |  |
| TTR | liver | pineal night | pineal day |
| EGFR | NA |  |  |
| IGF1R | prostate | placenta | whole blood |
| F2 | liver |  |  |
| MPO | bonemarrow | CD34+ | CD33+ myeloid |
| MET | NA |  |  |
| PARP1 | 721 B lymphoblasts | CD34+ | CD19+ B cells |
| PTGS2 | smooth muscle | bronchial epithelial cells | pancreatic islet |
| PPARA | NA |  |  |
| PTGS1 | smooth muscle | pineal night | cardiac myocytes |
| SLC5A2 | kidney |  |  |
| RXRA | CD71+ early erythroid | CD14+ monocytes | whole blood |
| NR1H4 | fetal liver | adrenal cortex |  |
| MMP8 | bonemarrow |  |  |
| BRAF | superior cervical ganglion |  |  |
| AURKA | 721 B lymphoblasts | CD71+ early erythroid | CD105+ endothelial |
| SGK1 | CD33+ myeloid | CD14+ monocytes | adrenal cortex |
| TNF | 721 B lymphoblasts | BDCA4+ dentritic cells | CD33+ myeloid |
| CCND1 | colorectal adenocarcinoma | prostate | lung |
| AGTR1 | adipocyte | adrenal cortex | adrenal gland |
| SIRT1 | CD19+ B cells | CD105+ endothelial | CD8+ T cells |
| CDK4 | 721 B lymphoblasts | CD105+ endothelial | CD34+ |
| GSTM1 | liver | adrenal gland |  |
| CASP1 | CD14+ monocytes | CD33+ myeloid | whole blood |
| CAPN1 | bronchial epithelial cells | lung | CD71+ early erythroid |
| NFKBIA | CD33+ myeloid | lung | adrenal cortex |
| RELA | lung |  |  |
| MAPK14 | CD33+ myeloid | CD56+ NK cells | whole blood |
| KDM5C | NA |  |  |
| HDAC6 | NA |  |  |
| EDNRB | retina | spinal cord | hypothalamus |
| CHEK1 | 721 B lymphoblasts | CD105+ endothelial | CD34+ |
| ADCY10 | NA |  |  |
| PIK3CA | CD8+ T cells | CD19+ B cells | 721 B lymphoblasts |
| MTOR | NA |  |  |
| ACE | NA |  |  |
| HLA-DRB1 | CD14+ monocytes | BDCA4+ dentritic cells | CD19+ B cells |
| IDH1 | prostate | adrenal cortex | adrenal gland |
| FLT1 | NA |  |  |
| HIF1A | bronchial epithelial cells | smooth muscle | cardiac myocytes |
| SHH | NA |  |  |
| XDH | NA |  |  |
| VDR | small intestine | colon |  |
